# Community- Weighted Mean Plant Traits Predict Small Scale Distribution of Insect Root Herbivore Abundance

**DOI:** 10.1371/journal.pone.0141148

**Published:** 2015-10-30

**Authors:** Ilja Sonnemann, Hans Pfestorf, Florian Jeltsch, Susanne Wurst

**Affiliations:** 1 Dahlem Centre of Plant Sciences, Freie Universitaet Berlin, Berlin, Germany; 2 Plant Ecology and Nature Conservation, Institute of Biochemistry and Biology, University of Potsdam, Potsdam, Germany; 3 Leibniz-Centre for Agricultural Landscape Research, ZALF, Müncheberg, Germany; 4 Berlin-Brandenburg Institute of Advanced Biodiversity Research (BBIB), Berlin, Germany; Estacion Experimental de Zonas Aridas - CSIC, SPAIN

## Abstract

Small scale distribution of insect root herbivores may promote plant species diversity by creating patches of different herbivore pressure. However, determinants of small scale distribution of insect root herbivores, and impact of land use intensity on their small scale distribution are largely unknown. We sampled insect root herbivores and measured vegetation parameters and soil water content along transects in grasslands of different management intensity in three regions in Germany. We calculated community-weighted mean plant traits to test whether the functional plant community composition determines the small scale distribution of insect root herbivores. To analyze spatial patterns in plant species and trait composition and insect root herbivore abundance we computed Mantel correlograms. Insect root herbivores mainly comprised click beetle (Coleoptera, Elateridae) larvae (43%) in the investigated grasslands. Total insect root herbivore numbers were positively related to community-weighted mean traits indicating high plant growth rates and biomass (specific leaf area, reproductive- and vegetative plant height), and negatively related to plant traits indicating poor tissue quality (leaf C/N ratio). Generalist Elaterid larvae, when analyzed independently, were also positively related to high plant growth rates and furthermore to root dry mass, but were not related to tissue quality. Insect root herbivore numbers were not related to plant cover, plant species richness and soil water content. Plant species composition and to a lesser extent plant trait composition displayed spatial autocorrelation, which was not influenced by land use intensity. Insect root herbivore abundance was not spatially autocorrelated. We conclude that in semi-natural grasslands with a high share of generalist insect root herbivores, insect root herbivores affiliate with large, fast growing plants, presumably because of availability of high quantities of food. Affiliation of insect root herbivores with large, fast growing plants may counteract dominance of those species, thus promoting plant diversity.

## Introduction

Insect root herbivores can alter plant community structure by affecting the competitive ability of individual plants [1]. Apart from overall abundance, small scale distribution of insect root herbivores may influence plant community structure [2]. Patches of different herbivore pressure in a site may add to the heterogeneity of growth conditions for plants and thereby to spatial structuring in vegetation composition at a local scale [3], thus promoting plant species diversity [4, 5].

Despite their potential impact on plant community structure, determinants of the small scale distribution of insect root herbivores are largely unknown [6]. Insect root herbivores mainly comprise larval stages. It is commonly thought that female insects lay their eggs in clusters where conditions are favorable at the time of oviposition [7, 8]. Subsequently, these patterns can be altered due to spatially and temporally changing mortality factors for eggs and first instar larvae [9]. For species with large, rather mobile larvae, movement of the larvae can create patterns with young individuals being more aggregated than medium aged larvae, and old individuals being regrouped again at attractive food sources [10, 11]. So far, small scale distribution of insect root herbivores has mostly been investigated for individual pest genera or species in monoculture crop fields. It can vary considerably between fields, and has been found to correlate with spatial variation of soil characteristics, soil moisture or host plant abundance [9, 12, 13]. Studies in diverse plant communities are only few [14, 15]. However, studying the determinants of insect root herbivores in diverse plant communities may contribute to our understanding of stabilizing mechanisms for plant species diversity (sensu [16]).

The spatial pattern of oviposition conditions, mortality factors and attractive food sources are determined by the spatial pattern of environmental factors within a site (from here on referred to as site heterogeneity). Thus, the degree of insect root herbivore aggregation should increase with increasing site heterogeneity. In semi-natural grasslands, site heterogeneity may be influenced by land use intensity. Land use intensification strongly impacts on grassland plant communities. This is reflected in the decrease of grassland plant species diversity within the last decades [17–19] and substantial differences in functional community composition along gradients of increasing land use intensity [20–22]. Moreover, land use regimes can also influence spatial patterns of plant species and life forms [23], and soil properties [23, 24]. Land use intensification in grasslands has been found to reduce spatial dependency of chemical soil properties, presumably due to fertilization and reduced plant species diversity [25]. From this, the authors inferred reduced habitat diversity for soil biota at the local scale with increasing land use intensity. Thus, besides direct negative effects of land use intensification on plant species diversity, land use intensification may also reduce the stabilizing effect of insect root herbivores on plant diversity, due to site homogenization and subsequent loss of patches of different herbivore pressure. Despite evidence for land use intensity impacts on site heterogeneity, potential impact of land use intensity on spatial pattern of insect root herbivores has not yet been studied.

Our study aimed at (i) identifying factors that determine the small scale distribution of insect root herbivores in grasslands and (ii) analyzing effects of land use intensity on the spatial pattern of insect root herbivore abundance. We sampled insect root herbivores and measured vegetation parameters and soil water content along transects in grasslands of different management intensity in three regions in Germany. We calculated community-weighted mean plant traits to test whether the functional plant community composition determines the small scale distribution of insect root herbivores. We computed Mantel correlograms to analyze spatial patterns in plant species and trait composition (as indication for site heterogeneity) and insect root herbivore abundance. All analyses on insect root herbivores were done on the ensemble of insect root herbivores as well as on Elaterid larvae separately. In European grasslands, Elaterid larvae mostly belong to the genus *Agriotes* [26, 27, Sonnemann personal observation], which are dominant generalist root herbivores [28–30], although other plant damaging genera are known as well [31]. Elaterid larvae also proved to be the most abundant insect root herbivores at the investigated sites. We expected root herbivore abundance to be positively correlated with (i) plant traits indicating high biomass and tissue quality, because this generally points to high food availability and (ii) soil water content, because this lowers the risk of desiccation of insect larvae. Additionally, we expected (iii) the spatial pattern of insect root herbivore abundance to mirror land use induced changes in site heterogeneity, with a homogenizing effect of land use intensification.

## Material and Methods

### Study design

The study was conducted within the frame of the Biodiversity Exploratories, at a total of 28 grassland sites that are located in three different regions in Germany with, 10, 9, and 9 sites in Schwäbische Alb, Hainich-Dün and Schorfheide-Chorin, respectively. Field work permits were issued by the Regierungspräsidium Tübingen for Schwäbische Alb, Thüringer Landesverwaltungsamt for Hainich-Dün, and Landesamt für Umwelt, Gesundheit und Verbraucherschutz Brandenburg for Schorfheide-Chorin (according to § 72 Nature Conservation Act of the federal state of Brandenburg). The three study regions span a latitudinal distance of 500 km. Their climate and soil conditions are described in detail in [32]. The grassland sites were selected to be comparable in terms of pedogenesis (cambisol and related soil units, [33]) but to differ in land use intensity in each region. A land use intensity index (LUI) was calculated for each site, according to [34], as LUI = F_*i*_/F_*R*_ + M_*i*_/M_*R*_ + G_*i*_/G_*R*_, where F_*i*_ quantifies the fertilizer application (0–94 kgN/ha), M_*i*_ the mowing frequency (0–3 cuts/year) and G_*i*_ the grazing intensity (9–1060 livestock units*d^-1^ha^-1^) at each site *i* in the years 2006–2010, with F_*R*_, M_*R*_ and G_*R*_ being their average value in the respective study region *R* (Schwäbische Alb, Hainich-Dün or Schorfheide-Chorin). Land use intensity indices ranged between 0.58 and 3.40, with low values indicating less and high values indicating more intensive management. Sampling took place along one 9 m transect at each site, with 30 evenly distanced samples per transect. Spatial grain (distance between samples) and spatial extent was chosen according to a rule by O’Neil et al. [35], who state that spatial grain should be maximally half the patch size of interest and spatial extent at least twice the size of the largest process under study. Root herbivore patches are expected to depend on the lateral influence zones of root systems of individual plants [2], which varies for grassland species between 0.6 and 1 m (compare [2, 36, 37]). This sets the grain to 0.3 m. Small scale soil heterogeneities can occur already on scales between 2 and 4 m [23, 38], justifying the extent of 9 m.

### Soil samples

Thirty soil cores of 5 cm diameter were taken to a depth of 6 cm and with a fixed distance of 0.3 m between cores at each site in October 2011. Large insect larvae were hand sorted from soil cores. Small insect larvae were extracted by subsequent heat extraction [39], in which soil fauna is forced to move out of the soil into a storage solution (ethylenglycol) by drying the soil core due to gradually increasing ambient temperature to 50°C over a period of 9 days. Insect larvae were stored in 70% ethanol until identification to family level [40, 41]. Families whose soil living species are mainly herbaceous or families with well-known root feeders in grasslands were categorized as root feeders. Number and identity of insect root herbivores was recorded for each soil core, and overall abundance of insect root herbivores and variance in insect root herbivore numbers in soil cores was calculated for each site. Soil core water content was determined for each soil core (including soil, roots, litter and small stones) as WC = ((fresh weight of soil core before heat extraction—dry weight of soil core after heat extraction) / dry weight of soil core after heat extraction) x 100. Roots were sorted from soil cores after heat extraction, washed, dried for 48 h at 56°C and weighed. Soil water holding capacity was determined from two additional soil cores per site, taken at both ends of the transect, as WHC = ((weight of water saturated soil core—dry weight of soil core (105°C for 26 h)) / dry weight of soil core) x 100. To saturate soil cores, intact soil cores were placed separately into mesh-bottomed beakers of 5.5 cm diameter and into water filled trays for 48 h.

### Vegetation records and plant species traits

Prior to soil sampling, vegetation was recorded from end of May to the beginning of July 2011 along the transects in adjacent plots of 0.3 x 0.3 m, centered on the position of each soil core. Vascular plant species were recorded, their ground cover was estimated in percent, and general features of the plant community, such as vegetation height and cover of bare soil were assessed in all plots. Shannon’s diversity index was calculated per plot as H’ = ∑pi x ln(pi), where pi is the share of ground cover represented by species i and is calculated as ground cover i / sum of ground cover over all species. Additionally, Evenness was calculated as J’ = H’/ln(S), where S equals species richness.

Ten plant traits (Table 1) that are (i) sensitive to differences in land use intensity (see [21] and references therein) and (ii) possible predictors of root herbivore abundance were compiled to describe functional composition of plant communities. For the 84 most common species in our vegetation records, six shoot traits and root C/N ratios were measured in the field in June 2011 on different sites in the Haininch Dün region. Since excavation of whole root systems including fine roots was not possible in sufficient replication [42–47] for the numerous plant species, we used shoot traits as proxies for root trait differences [48]. The selected shoot traits can, besides their well-known implications for competitive vigor and stress tolerance, serve as proxies for herbivore deterrence, root palatability and root morphology (see Table 1 and references therein). Traits for a respective plant species were recorded on sampling sites representing the grassland plant society in which the species typically occurs. All samples of a species were recorded within one population. Shoot traits and leaf samples were attained according to a standardized protocol [49]: For each species we sampled 20 young, but fully expanded leafs, including petiole or rachis, from undamaged adult plants. Samples were sealed in plastic bags and stored in a cool box until further processing. Leaves were rehydrated and fresh mass was measured before leaf area was assessed by using a standard flatbed scanner (resolution 300 dpi) and the software Lafore (https://www.uni-oldenburg.de/en/biology/landeco/download-and-service/software/lafore/). Leaf thickness was measured at 2 points on the leave, avoiding midribs, using a high precision caliper (+/- 0.01 mm). Leaf dry mass was determined after drying at 60°C for 48 h. Specific leaf area was calculated as SLA = area / dry mass. Leaf dry matter content was calculated as LDMC = dry mass / fresh mass. Leaf density was calculated as dry mass * area^-1^ * leaf thickness^-1^. Vegetative plant height was determined as VH = height of foliage, and reproductive plant height as RH = height of inflorescence, for 30 undamaged adult plants. To assess root C/N ratios, root systems of five randomly selected individuals per species were excavated as completely as possible. Leaf and root C and N contents were determined by means of combustion (elemental analyzer EuroEA, HEKATech GmbH, Germany) from one homogenized leaf- and one homogenized root sample per species, respectively. If available, field measurements were complemented with trait values form the LEDA traitbase [50] for the remaining, less common plant species in the species list. The trait ‘vegetative spread’ was derived from the database Clo-Pla 3 [51] and was treated as a continuous variable by calculating the mean value of classes provided in the original database. The trait ‘root density in soil’ (in the whole soil profile and in the 0–0.2 m topsoil) was derived by scanning highly accurate drawings of whole root systems, which are depicted in the root atlas of Kutschera and Lichtenegger [36, 37] and originate from excavations of typical individuals of plant species. Adobe Photoshop CS4 (version 11.02) was then used to determine the proportion of fill [%] in each image. Community-weighted mean traits were calculated per 0.3 x 0.3 m plots as follows: trait_cmeans_ = ∑ci x trait_i_, where ci is the relative cover contribution of species i to the community and trait_i_ is the trait value of species i (see also [20]). Plots in which species with attributed trait values collectively made up less than 80% of total cover (see Table 1) were omitted from subsequent analyses on community-weighted mean traits [52].

**Table 1:** List of plant functional traits used as predictors in generalized mixed effects models, with abbreviations, data source and relevance for explaining root herbivore abundance.

Trait	Abbre-viation	Unit	Source	Relevance/ link to root traits	% Plots covered
Specific leaf area	SLA	mm^2^ mg^-1^	o.m., LEDA	rel. growth rate [53], herbivore deterrence [43, 53, 54]	100
Leaf dry matter content	LDMC	mg g^-1^	o.m., LEDA	rel. growth rate [53], herbivore deterrence [43, 53, 54]	100
Leaf density		mg mm^-3^	o.m.	palatability [42, 43]	89.3
Vegetative plant height	VH	m	o.m., LEDA	biomass, rooting depth and width [55]	99.7
Reproductive plant height	RH	m	o.m., LEDA	biomass, rooting depth and width [55]	100
Leaf C/N		-	o.m.	food quality [42, 43]	92.0
Root C/N		-	o.m.	food quality [42, 43]	91.0
Vegetative spread		m	Clo-Pla 3	root foraging [56]	100
Root density in soil		%	ref 1,2	food quantity	77.1
Root density in topsoil (0–0.2 m)		%	ref 1,2	food quantity	77.1

o.m. = own (field) measurement

### Statistical analyses

We used linear mixed effects models to determine factors that influence insect root herbivore abundance. Spatial patterns of insect root herbivore abundance and plant composition were analyzed by Mantel correlogram analysis. Impact of land use intensity on spatial patterns and on variance in insect root herbivore numbers in soil cores was again analysed with linear mixed effect models and linear models, respectively. All statistical analyses were conducted using the software R, version 3.0.3 [57].

To determine factors that influence total insect root herbivore numbers and numbers of the most abundant insect root herbivore family in individual soil samples, we fit generalized linear mixed effect models (GLMM), with community-weighted mean traits as well as soil core- and vegetation parameters as predictors, assuming Poisson distribution of errors. To enable ranking of predictors according to effect strength, predictor values were z-standardised, and one separate model was fit for each predictor. As GLMM analysis may be biased by an underlying spatial structure of the response variable [58], three sites at which spatial autocorrelation of root herbivore abundances was indicated by significant global Moran’s I were excluded from the analysis. Data for cover of bare soil was log transformed prior to analysis to achieve normal distribution in model residuals. Our initial data-frame appeared to be severely zero-inflated, causing over-dispersion and non-normality of model residuals. As this problem was not accountable for with currently available methods [59], we reduced zeroes by summing up root herbivore abundances and averaging predictor variables over three adjacent samples along transects, resulting in ten values for root herbivore abundance and each predictor per transect. We calculated conditional R^2^ as proposed by Nakagawa and Schielzeth [60] as a measure of the goodness of fit.

Spatial patterns of insect root herbivore abundance, plant species composition and trait composition (composition of community-weighted mean traits) were analyzed within each transect by Mantel correlogram analysis. Distance classes along transects were calculated using the Sturges equation [61] resulting in 10 distance classes (0–0.72 m, 0.72–1.56 m, 1.56–2.40 m, 2.40–3.24 m, 3.24–4.08 m, 4.08–4.92 m, 4.92–5.76 m, 5.76–6.60 m, 6.60–7.44 m, 7.44–8.28 m). Mantel correlograms were based on Bray-Curtis dissimilarities for plant species composition and on Euclidean distances for trait composition. We considered a parameter to be spatially autocorrelated if Mantel correlogram analysis revealed significant correlation coefficients for one or several distance classes. We considered a parameter to be spatially aggregated/patchily distributed, with abundances decreasing gradually from the centre of patches, if the analysis produced significant positive coefficients (similarity of samples) for short distances, which became zero or negative with increasing distances [61, 62]. To test whether spatial patterns depend on land use intensity and soil conditions, linear mixed effect models [63] were fit for parameters that displayed spatial autocorrelation or patchiness, with Mantel correlation coefficients as response variables and LUI, soil water holding capacity and distance classes as predictors, allowing for one way interactions between predictors. Effects of land use intensity on overall abundance of insect root herbivores and variance in insect root herbivore numbers in soil cores in each site were analyzed using linear models.

Pseudo-replication was corrected for in all mixed effects models by including site identity as a random factor working on the intercept. Region could not be treated as random factor due to small number of factor levels (3) and was thus included as fixed factor. However, as we were not interested in regional differences, intercepts for the different regions were averaged for result presentation. Distribution of errors and homogeneity of variances for mixed effect models was checked using graphical residual diagnostics (QQ plot, plot of residuals vs. fitted values and a histogram of residuals). All models were simplified in a backwards stepwise manner until the minimum adequate model remained. A model term was treated as non-significant and removed from the initial model if parametric bootstraps (R package pbkrtest; see [64]) yielded p values >0.05.

## Results

A total of 551 insect root herbivores, belonging to eight families (Table 2) from two different orders (Coleoptera, Diptera), were sampled in the grasslands, with an average of 20 (sd +/- 12) individuals per site. Numbers of insect root herbivores per sample (soil core) ranged from 0 to 22 individuals, and variance in numbers at sample points was on average 1.5 (sd +/- 3.0) per site. Larvae of the click beetle (Elateridae) were the most abundant insect root herbivores (43% of all individuals) in the investigated grasslands. A list of all 191 recorded plant taxa with all available trait values is given in supplementary material (S1 Table). Average soil water holding capacity at sites was 63 (sd +/- 26) % soil dry weight.

**Table 2:** Root feeding insect s.

Order	Family	Share of total numbersof individuals [%]
Coleoptera	Byrrhiidae	0.2
Coleoptera	Chrysomelidae	4.5
Coleoptera	Curculionidae	18.3
Coleoptera	Elateridae	43.0
Coleoptera	Scarabaeidae	0.2
Diptera	Cecidomyiidae	29.6
Diptera	Stratiomyidae	0.4
Diptera	Tipulidae	3.8

GLMM analysis revealed that total insect root herbivore numbers in individual soil samples were positively related to community-weighted mean traits indicating high plant growth rates and biomass (Table 3), namely to specific leaf area and reproductive- and vegetative plant height. Insect root herbivore numbers were negatively related to plant traits indicating slow plant growth and poor tissue quality, namely to leaf dry matter content and leaf C/N ratio, respectively. Numbers of Elaterid larvae in individual soil samples were also positively related to traits indicating high plant growth rates and biomass, and furthermore to root dry mass in soil cores (Table 4). Contrary to total insect root herbivore numbers, Elaterid larvae numbers were not related to plant tissue quality. Total insect root herbivore and Elaterid larvae numbers were not related to the remaining community-weighted mean traits, soil core- and plant community parameters.

**Table 3:** Mean values (calculated from original values) of and effects (generalized linear mixed effect models, calculated from z-standardized predictors) of community-weighted mean traits, soil core- and vegetation parameters on insect root herbivore abundance.

Predictor	Mean(sd)	Χ^2^	p	Mean intercept	Slope	R^2^
*Community-weighted mean traits*						
Specific leaf area [mm^2^ mg^-1^]	20.1 (2.8)	11.70	**	0.365	0.283	0.39
Leaf dry matter content [mg g^-1^]	316 (44)	6.57	*	0.385	-0.197	0.30
Leaf density [mg mm^-3^]	0.29 (0.04)	19.87	ns	0.373	-0.177	0.34
Vegetative plant height [m]	0.24 (0.08)	5.17	*	0.384	0.153	0.30
Reproductive plant height [m]	0.39 (0.10)	8.09	**	0.383	0.193	0.31
Leaf C/N	19.6 (2.7)	14.67	**	0.370	-0.260	0.36
Root C/N	36.6 (6.2)	4.55	ns	0.373	-0.113	0.36
Vegetative spread [m]	0.06 (0.03)	0.47	ns	0.381	0.044	0.33
Root density in soil [%]	0.07 (0.02)	67.51	ns	0.417	0.017	0.34
Root density in top soil [%]	0.12 (0.03)	67.71	ns	0.415	0.040	0.35
*Soil core parameters*						
Water content [%DW]	32.7 (18.8)	1.45	ns	0.371	0.277	0.31
Root dry mass [g soil core^-1^]	0.6 (0.5)	0.33	ns	0.383	-0.031	0.32
*Plant community parameters*						
Sum of plant cover all species [%]	95.6 (31.1)	1.07	ns	0.384	0.083	0.32
Cover of bare soil [%]	9.7 (13.1)	0.97	ns	0.382	0.070	0.33
Species richness [no 0.09m^-2^]	15.1 (7.7)	3.08	ns	0.378	-0.184	0.34
Shannons H’	2.0 (0.5)	1.20	ns	0.380	-0.094	0.33
Eveness	0.8 (0.1)	0.13	ns	0.381	-0.023	0.33

Significance levels;

**: p<0.01,

*: p<0.05,

ns: not significant p>0.05; DW: dry weight

**Table 4:** Effects (generalized linear mixed effect models, calculated from z-standardized predictors) of community-weighted mean traits, soil core- and vegetation parameters on abundance of Elaterid larvae.

Predictor	Χ^2^	p	Mean intercept	Slope	R^2^
*Community-weighted mean traits*					
Specific leaf area	11.25	**	-0.614	0.425	0.45
Leaf dry matter content	2.18	ns	-0.564	-0.169	0.34
Leaf density	27.57	*	-0.581	-0.356	0.36
Vegetative plant height	8.43	**	-0.580	0.292	0.33
Reproductive plant height	9.45	**	-0.574	0.296	0.35
Leaf C/N	14.14	ns	-0.584	-0.328	0.41
Root C/N	6.50	ns	-0.578	-0.085	0.36
Vegetative spread	0.33	ns	-0.580	-0.055	0.34
Root density in soil	56.79	ns	-0.572	0.277	0.44
Root density in top soil	54.93	ns	-0.559	0.220	0.41
*Soil core parameters*					
Soil water content	1.43	ns	-0.648	0.416	0.35
Root dry mass	8.37	**	-0.591	0.214	0.38
*Vegetation parameters*					
Sum of plant cover all species	0.19	ns	-0.582	0.050	0.35
Cover of bare soil	0.10	ns	-0.580	0.031	0.35
Species richness	0.13	ns	-0.579	-0.055	0.35
Shannons H’	0.01	ns	-0.581	-0.010	0.35
Eveness	0.05	ns	-0.583	-0.022	0.36

Significance levels;

**: p<0.01,

*: p<0.05,

ns: not significant p>0.05

**Fig 1:**
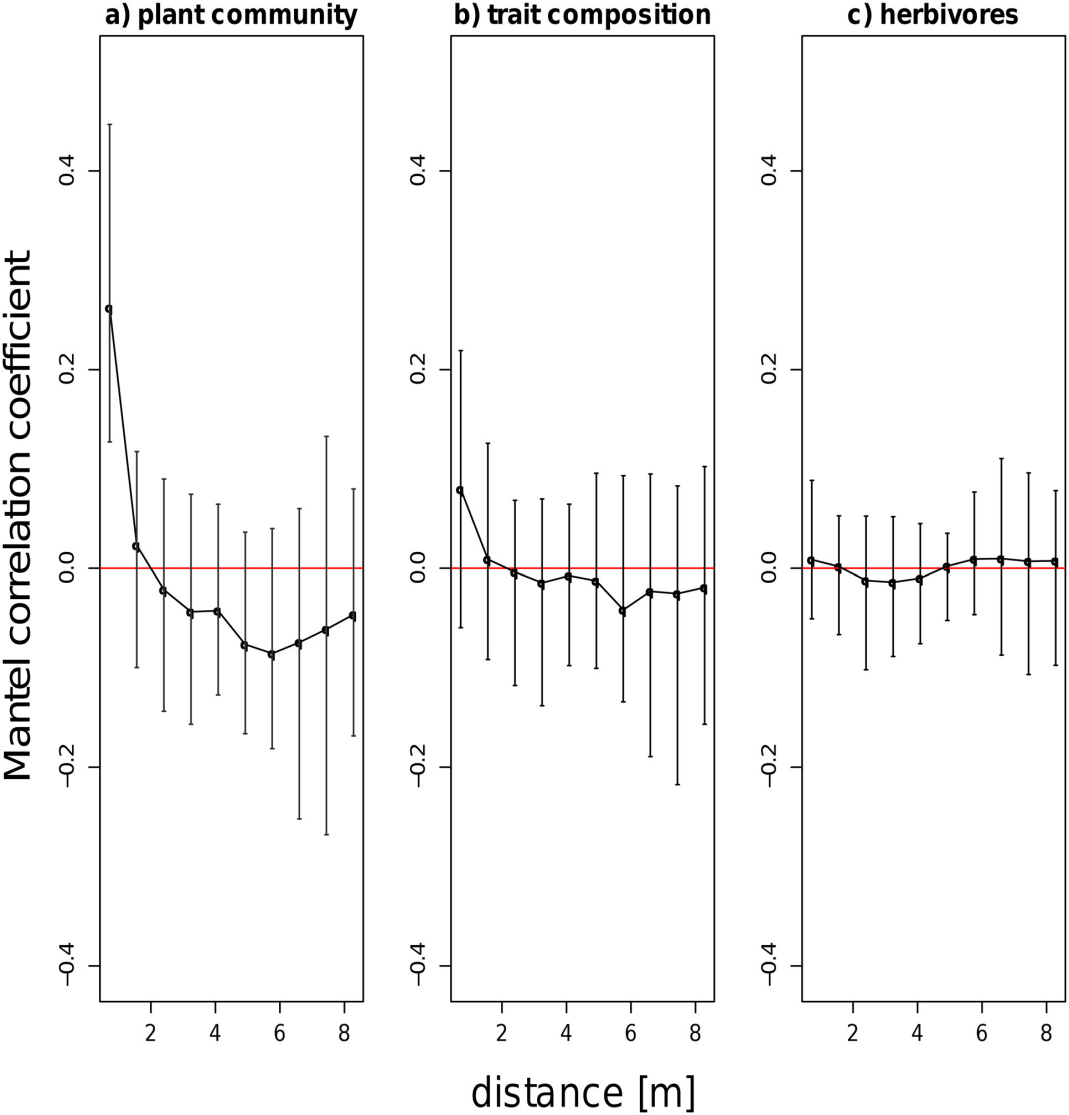
Average mantel correlation coefficients. over all investigated sites with standard deviation for a) plant communities, b) trait composition and c) insect root herbivore abundance. Mantel correlograms for individual sites are given in the supplementary material.

Plant species composition showed significant Mantel correlations at all sites (for comparison of Mantel correlograms see supplementary material, S1 Fig). In general, we detected significant positive correlation coefficients in the shortest distance class. Coefficients then decreased and became negative in larger distance classes, pointing to patchiness of the vegetation. Reoccurring patterns of increasing and decreasing coefficients with increasing distance classes indicated presence of patches with different spatial extent at site HEG 43. Trait composition was less clearly spatially patterned. Although we detected evidence for a patchy distribution comparable to the spatial patterns of plant species composition at half (14) of the sites, significant positive correlation coefficients in the short distance classes, were clearly lower here (see Fig 1) and correlation coefficients were not significant for larger distance classes. In eight sites we did not detect any significant Mantel correlations for trait composition. Total insect root herbivore and Elaterid larvae abundance was not spatially autocorrelated at the majority of sites (23 out of 28 sites). Significant Mantel correlations were detected at only five sites, and in varying distance classes. Neither Mantel correlation coefficients for plant species composition nor trait composition were related to land use intensity or soil water holding capacity (supplementary material, S2 Table), indicating that their spatial pattern was not affected by these factors. As total insect root herbivore and Elaterid larvae abundance did not display spatial autocorrelation, Mantel coefficients of these parameters were not tested for influence of land use intensity or soil water holding capacity. Overall abundance of insect root herbivores and variance in insect root herbivore numbers in soil cores in each site were also not affected by land use intensity (R^2^ = 0.03; p>0.05 and R^2^ = 0.02; p>0.05, respectively).

## Discussion

The presented study aimed at (i) identifying factors that determine the small scale distribution of insect root herbivores in grasslands and (ii) analyzing effects of land use intensity on the spatial pattern of insect root herbivore abundance. As hypothesized, insect root herbivore abundance was positively related to plant traits indicating high growth rate and biomass, but negatively correlated to traits indicating slow growth and poor tissue quality. Contrarily to expectations, insect root herbivore abundance was not related to soil core water content. Plant species and trait compositions displayed spatial autocorrelation, with plant species composition being patchy. However, insect root herbivore abundance did not mirror these patterns, as it was not spatially autocorrelated. Also, neither spatial pattern of plant species nor trait compositions was influenced by land use intensity.

Affiliation of insect root herbivores with plants of high biomass, growth rate and tissue quality supports our hypothesis that they are found in spots of high food availability. Insect root herbivore populations in our study were dominated by Elaterid larvae, which are generalist root feeders [26]. Generalist Elaterid larvae have been found to display food preferences that were ascribed to food quality [65], and high plant growth rates may be related to low plant defenses [66]. However, in our study, analyses regarding Elaterid larvae separately suggest that food availability for Elaterid larvae is rather driven by food quantity than by food quality, as abundance of Elaterid larvae was additionally correlated with root dry mass, but was not affected by plant tissue C/N ratios. In accordance with a quantity driven food availability, horizontal migration [67] as well as growth of Elaterid larvae [30] has been found to depend on root biomass. Though migration of soil living insect larvae was found to depend on soil water content [68, 69], abundance of insect root herbivores was not influenced by soil core water content in our study. Soil core water content varied by 19% over all samples, however, within site variation was on average only 4% (data not shown), and thus apparently not high enough to influence insect root herbivore abundance.

Insect root herbivore abundance was not spatially patterned at the scale investigated in our study. In accordance, [14] found no spatial autocorrelation for larvae of several herbivorous insect species, including Elateridae, in grasslands sampled at distances from 0.8–40 m. Minimal sampling distance (0.3 m) was chosen in relation to the lateral spread of individual root systems in our study. However, as distribution of abundance was likely driven by food quantity for the majority of insect root herbivores, and root biomass may vary considerably between different areas within a root system (compare [36, 37, 67]), spatial patterning of insect root herbivore abundance may only emerge below distances of 0.3 m. Though insect root herbivore abundance was not spatially patterned in a sense that abundances decreased gradually from patch centers (as indicated by Mantel correlogram analyses), affiliation with large, fast growing plants still suggests that abundances are higher at the location of these plants compared to the remaining area of the sites. Affiliation of insect root herbivores with large, fast growing plants may counteract dominance of those species, thus promoting plant diversity. Land use practices like fertilization and cutting may enhance the abundance of large, fast growing plants [70]. However, overall insect root herbivore abundance did not increase with increasing land use intensity, and distribution of insect root herbivores remained constant, as indicated by a lack of impact of land use intensity on variance in herbivore numbers at sample points within sites. This suggests that enhanced root herbivore pressure persisted for a given number of large, fast growing plant individuals, and that the potential stabilizing effect of insect root herbivores on plant diversity was not reduced by land use intensification over the range of land use intensities investigated in our study.

Plant species and trait compositions were spatially patterned, with plant species composition being patchy. Despite affiliation of insect root herbivores with certain plant traits, spatial pattern of plant trait composition as well as of single plant traits (tested for SLA as the most influential trait, data not shown) was apparently too weak (as indicated by low Mantel correlation coefficients) to determine those of insect root herbivores. Although plant species richness decreased with increasing land use intensity in our study (R^2^ = 0.39; p<0.001), land use intensity did not affect the spatial pattern of plant species and trait composition. In accordance, [25] did not find the expected homogenizing effect of land use intensity, especially fertilization, on microbiological soil properties in managed grasslands. On the other hand, [23] found the spatial pattern of plant live forms and individual grassland plant species to be influenced by grazing regime. Additional analyses, in which land use intensity was replaced by individual land use components (fertilization, mowing, grazing; effects = n.s., data not shown), ruled out that effects of individual land use components offset each other in our study. Differences in findings of [23] and our study may rather be explained by resolution in land use categorization (presence/absence of management practices vs. intensity gradient).

To summarize, our study revealed that, in semi-natural grasslands with a high share of generalist insect root herbivores, insect root herbivores affiliate with large, fast growing plants, presumably because of availability of high quantities of food. Affiliation of insect root herbivores with large, fast growing plants may counteract dominance of those species, thus promoting plant diversity. This potential stabilizing effect of insect root herbivores on plant diversity was not reduced by land use intensification over the range of land use intensities investigated in our study. Insect root herbivore abundance was not spatially patterned at the scale investigated in our study. A food quantity driven distribution suggests spatial patterning within the root system of individual plants. Spatial patterning of insect root herbivores on that scale remains to be investigated.

## Supporting Information

S1 Fig Mantel correlation coefficients in relation to distance classes along transects for plant species and trait composition as well as for abundance of all insect root herbivores and Elaterid larvae only. Filled symbols indicate significant correlations at p < 0.05. AEG: grassland sites in Schwäbische Alb, HEG: grassland sites in Hainich Dün, SEG: grassland sites in Schorfheide Chorin. Numbers in site IDs indicate site identity among 50 sites that are present in each region (compare [32]); they are not indicative for land use intensity or any other site traits.
(PDF)

S1 Table Trait values for identified plant taxa. Values printed in italics stem from the LEDA traitbase.
(XLSX)

S2 Table Effects (linear mixed effects models) of land use intensity (LUI), soil water holding capacity (WHC) and distance on spatial pattern (Mantel correlation coefficients, MCC) of plant species and trait composition. (DOCX)
