# Supplementary material for: Community- Weighted Mean Plant Traits Predict Small Scale Distribution of Insect Root Herbivore Abundance
Source: PLoS One. 2015 Oct 30;10(10):e0141148. doi: 10.1371/journal.pone.0141148 (PMC4627808; doi:10.1371/journal.pone.0141148)
Supplement: S1 Fig — Filled symbols indicate significant correlations at p < 0.05. AEG: grassland sites in Schwäbische Alb, HEG: grassland sites in Hainich Dün, SEG: grassland sites in Schorfheide Chorin. Numbers in site IDs indicate site identity among 50 sites that are present in each region (compare [32]); they are not indicative for land use intensity or any other site traits. (PDF) [file pone.0141148.s001.pdf]

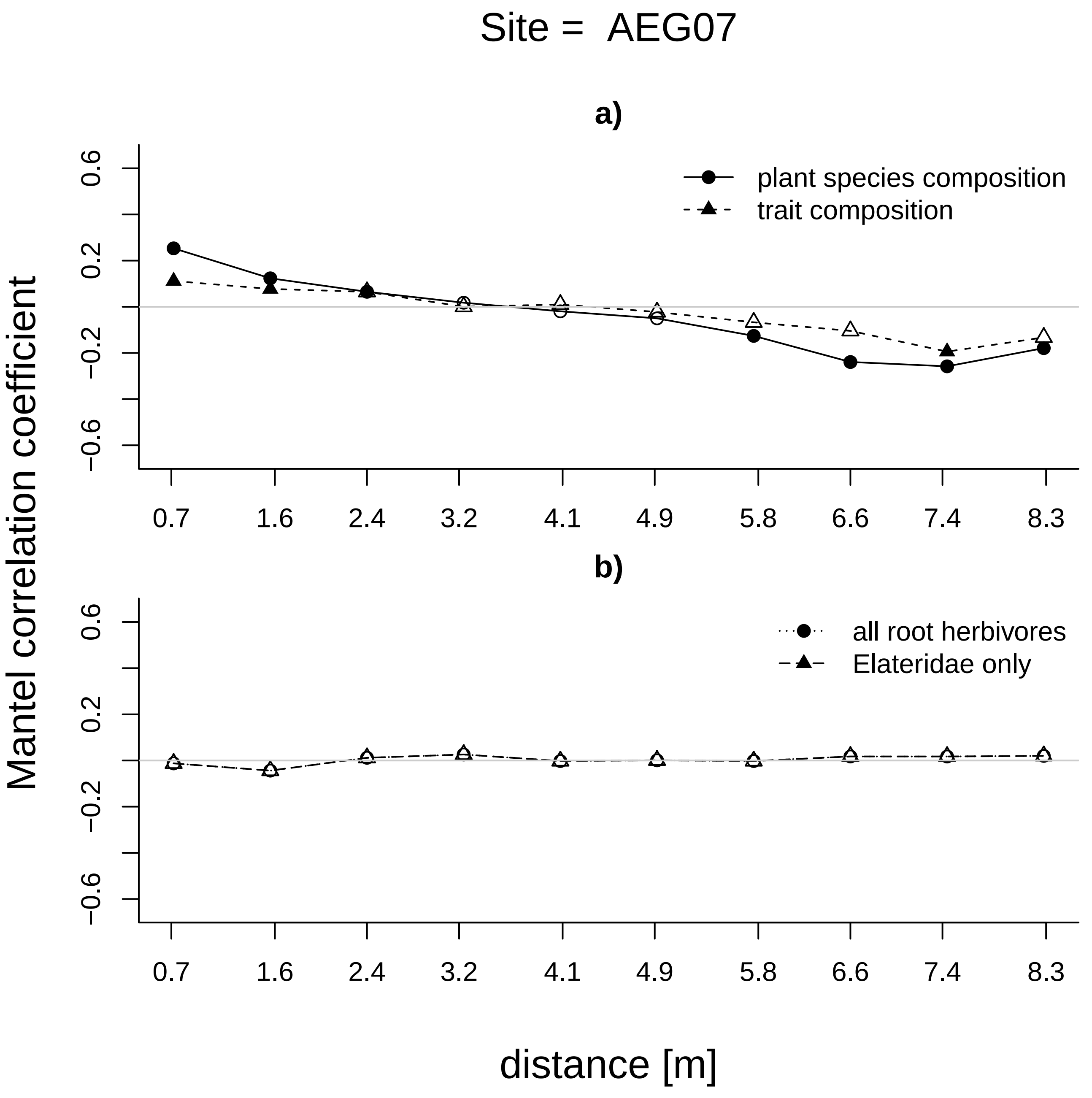

Site = AEG07
a)
plant species composition
trait composition
0.6
0.2
−0.2
−0.6
0.7
1.6
2.4
3.2
4.1
4.9
5.8
6.6
7.4
8.3
b)
all root herbivores
Elateridae only
0.6
0.2
−0.2
−0.6
0.7
1.6
2.4
3.2
4.1
4.9
5.8
6.6
7.4
8.3
Mantel correlation coefficient
distance [m]

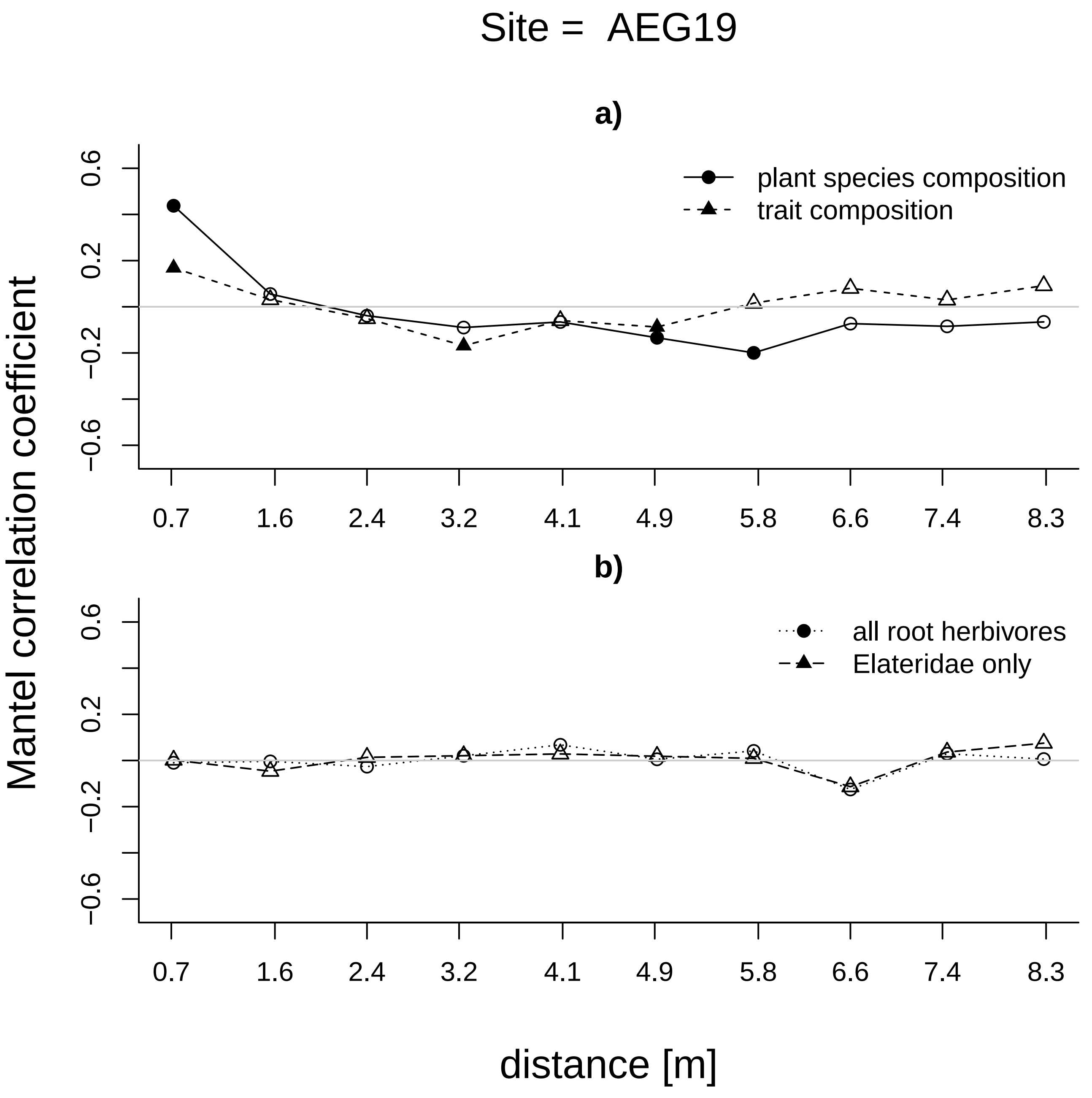
Site = AEG19
a)
plant species composition
trait composition
b)
all root herbivores
Elateridae only
Mantel correlation coefficient
0.6
0.2
−0.2
−0.6
0.7
1.6
2.4
3.2
4.1
4.9
5.8
6.6
7.4
8.3
distance [m]

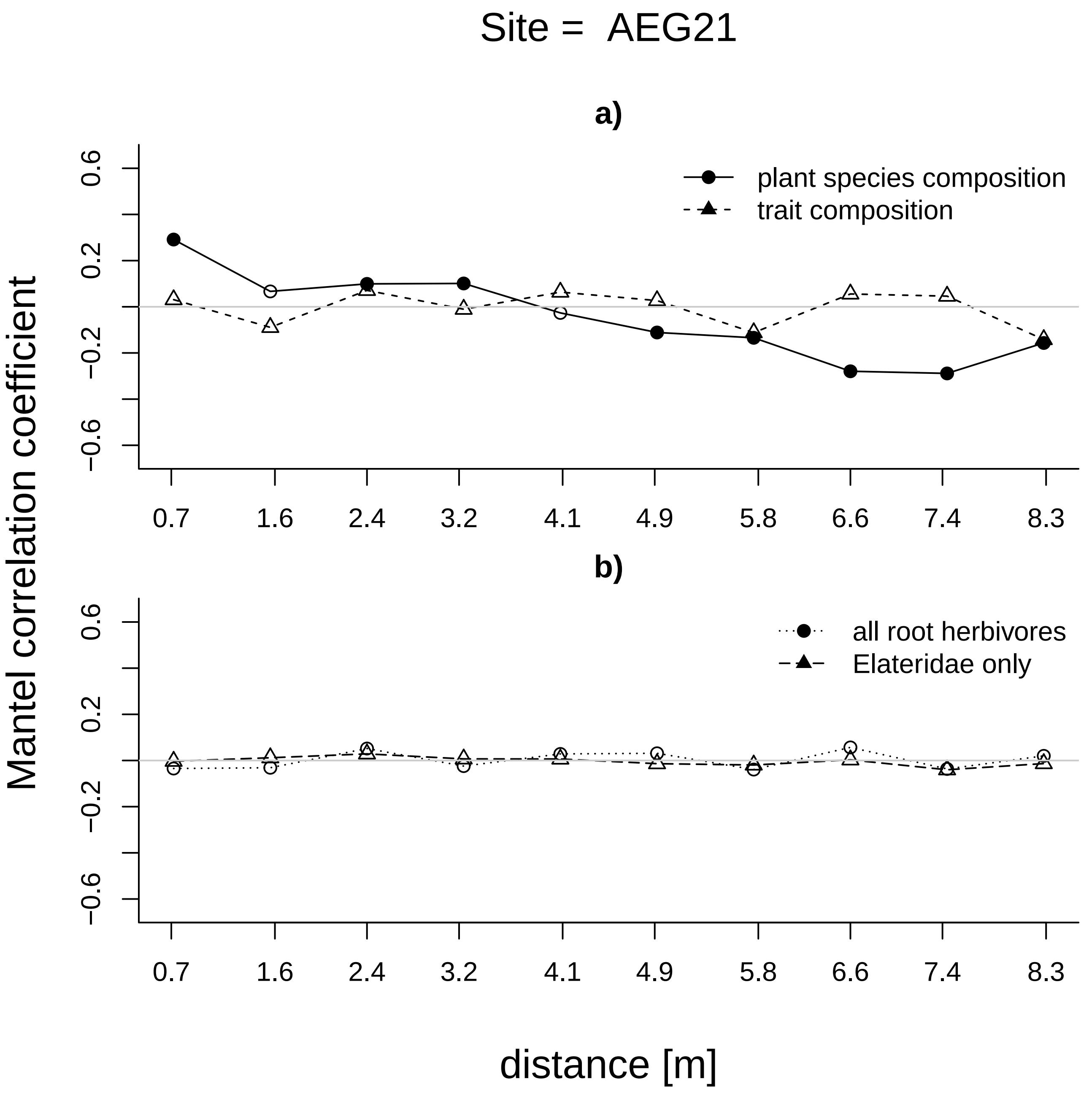
Site = AEG21
a)
plant species composition
trait composition
b)
all root herbivores
Elateridae only
Mantel correlation coefficient
distance [m]
0.6
0.2
−0.2
−0.6
0.7
1.6
2.4
3.2
4.1
4.9
5.8
6.6
7.4
8.3

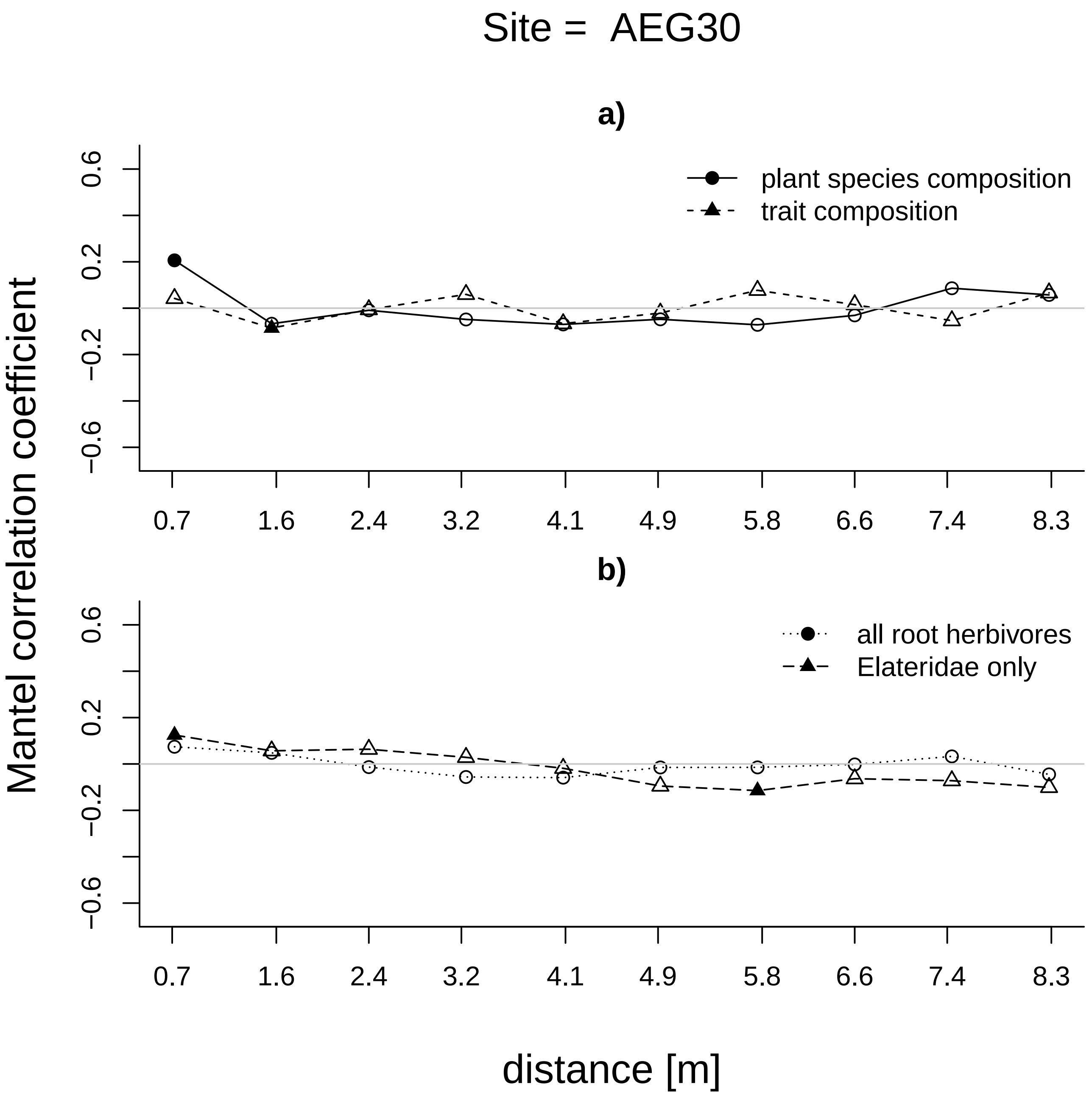

Site = AEG30
a)
plant species composition
trait composition
Mantel correlation coefficient
0.6
0.2
−0.2
−0.6
0.7
1.6
2.4
3.2
4.1
4.9
5.8
6.6
7.4
8.3
b)
all root herbivores
Elateridae only
distance [m]

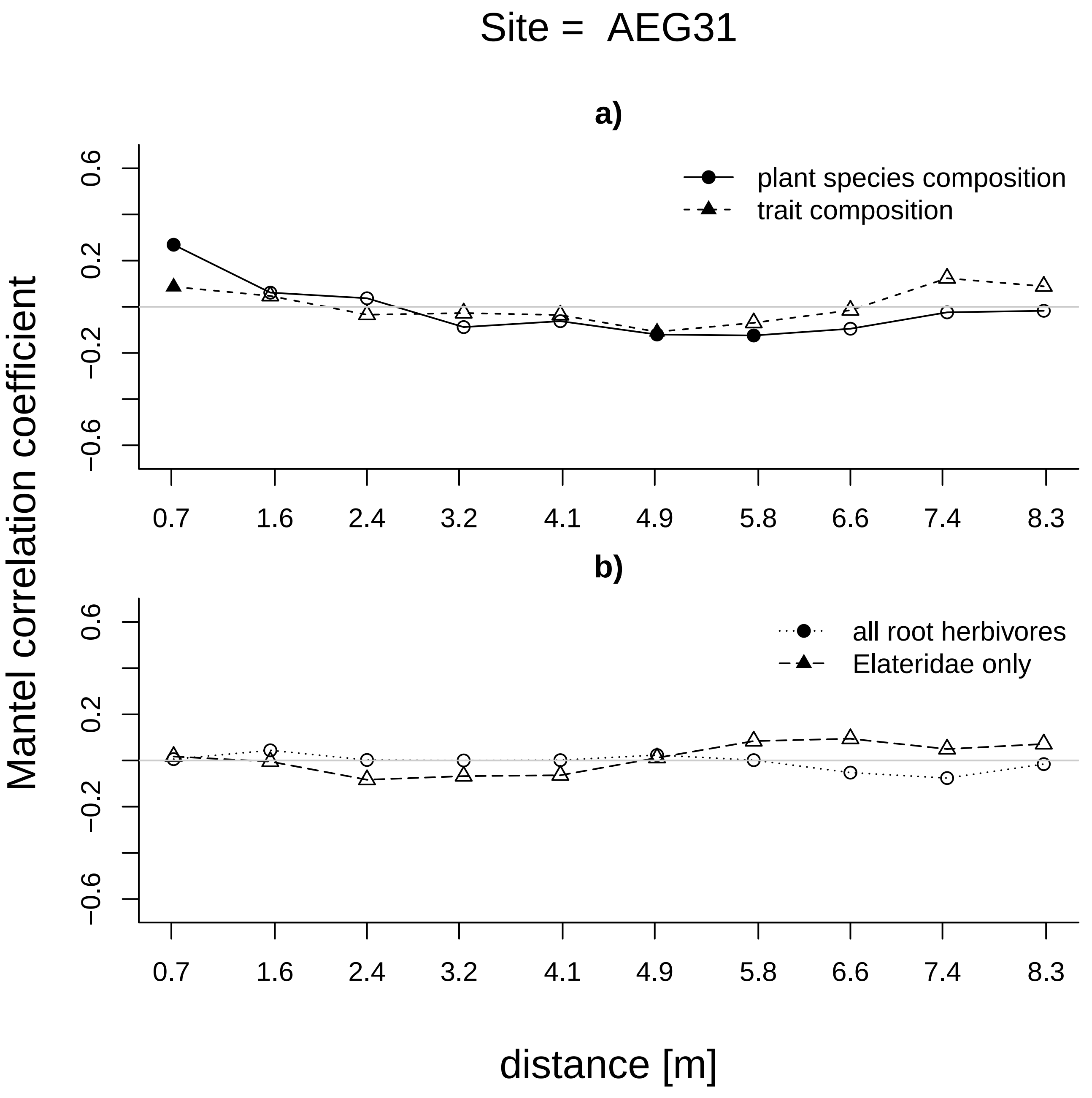
Site = AEG31
a)
plant species composition
trait composition
b)
all root herbivores
Elateridae only
Mantel correlation coefficient
0.6
0.2
−0.2
−0.6
0.7
1.6
2.4
3.2
4.1
4.9
5.8
6.6
7.4
8.3
distance [m]

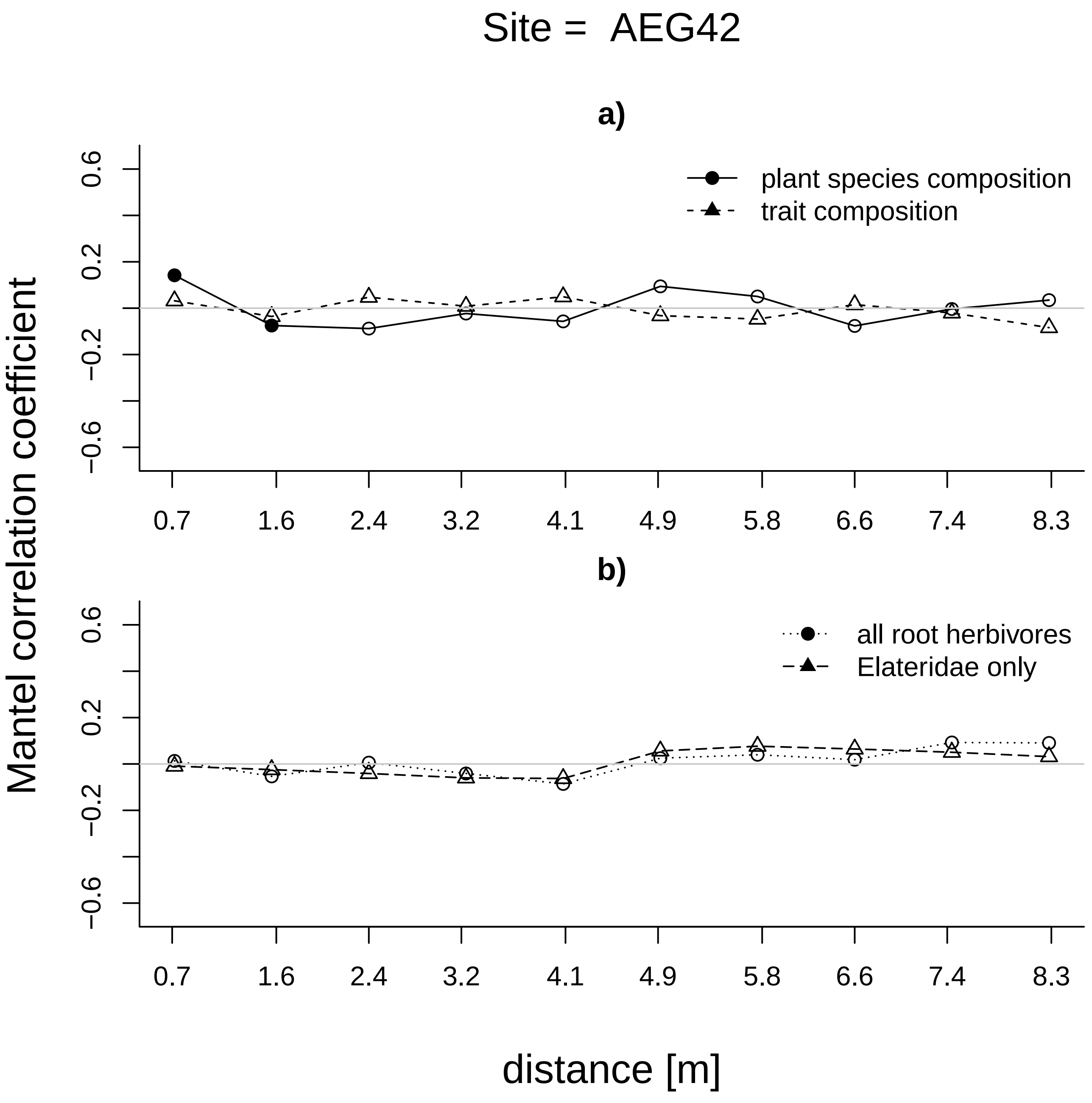

Site = AEG42
a)
plant species composition
trait composition
b)
all root herbivores
Elateridae only
Mantel correlation coefficient
distance [m]
0.6
0.2
−0.2
−0.6
0.7
1.6
2.4
3.2
4.1
4.9
5.8
6.6
7.4
8.3

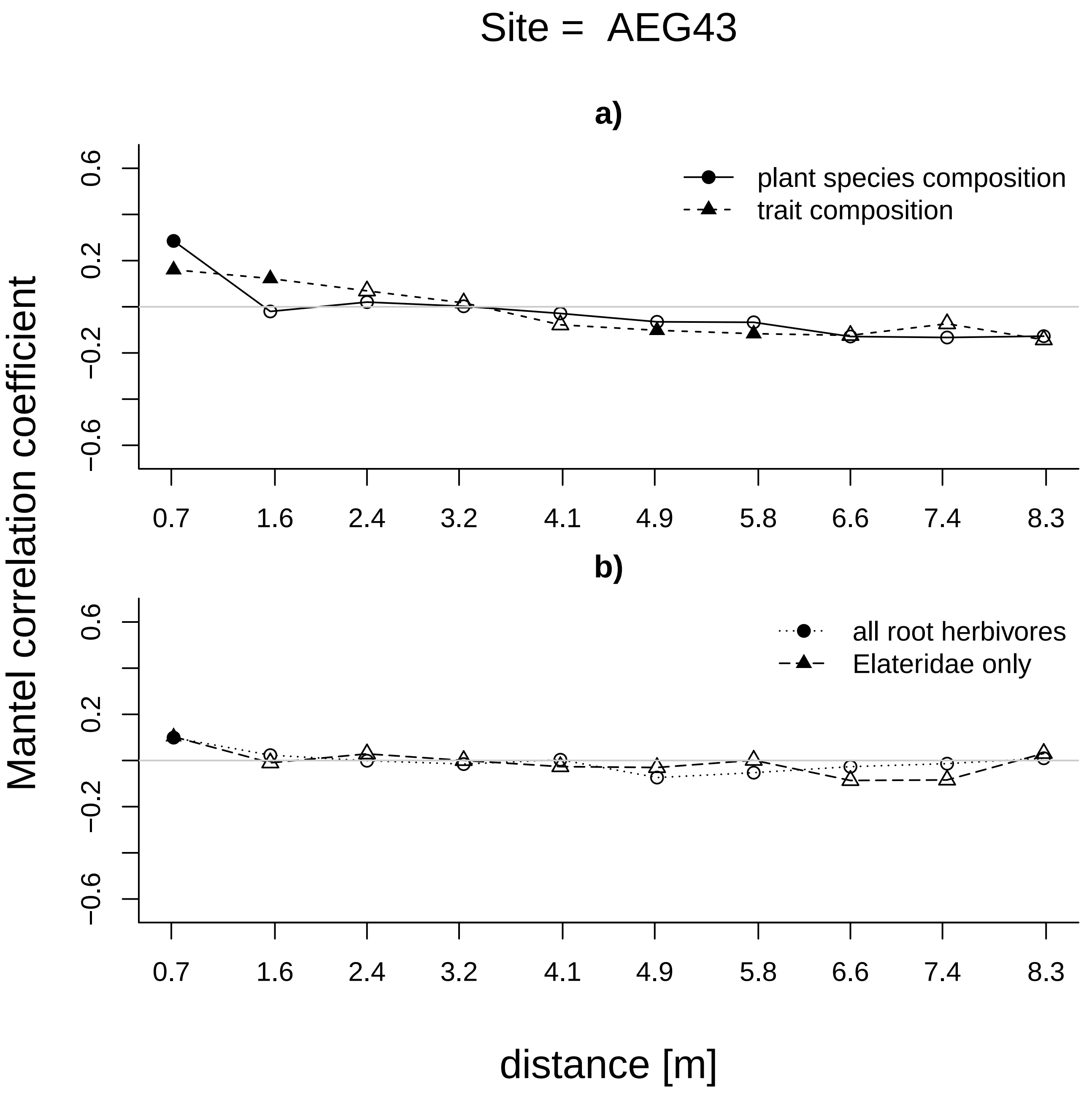

Site = AEG43
a)
plant species composition
trait composition
Mantel correlation coefficient
0.6
0.2
−0.2
−0.6
0.7
1.6
2.4
3.2
4.1
4.9
5.8
6.6
7.4
8.3
b)
all root herbivores
Elateridae only
distance [m]

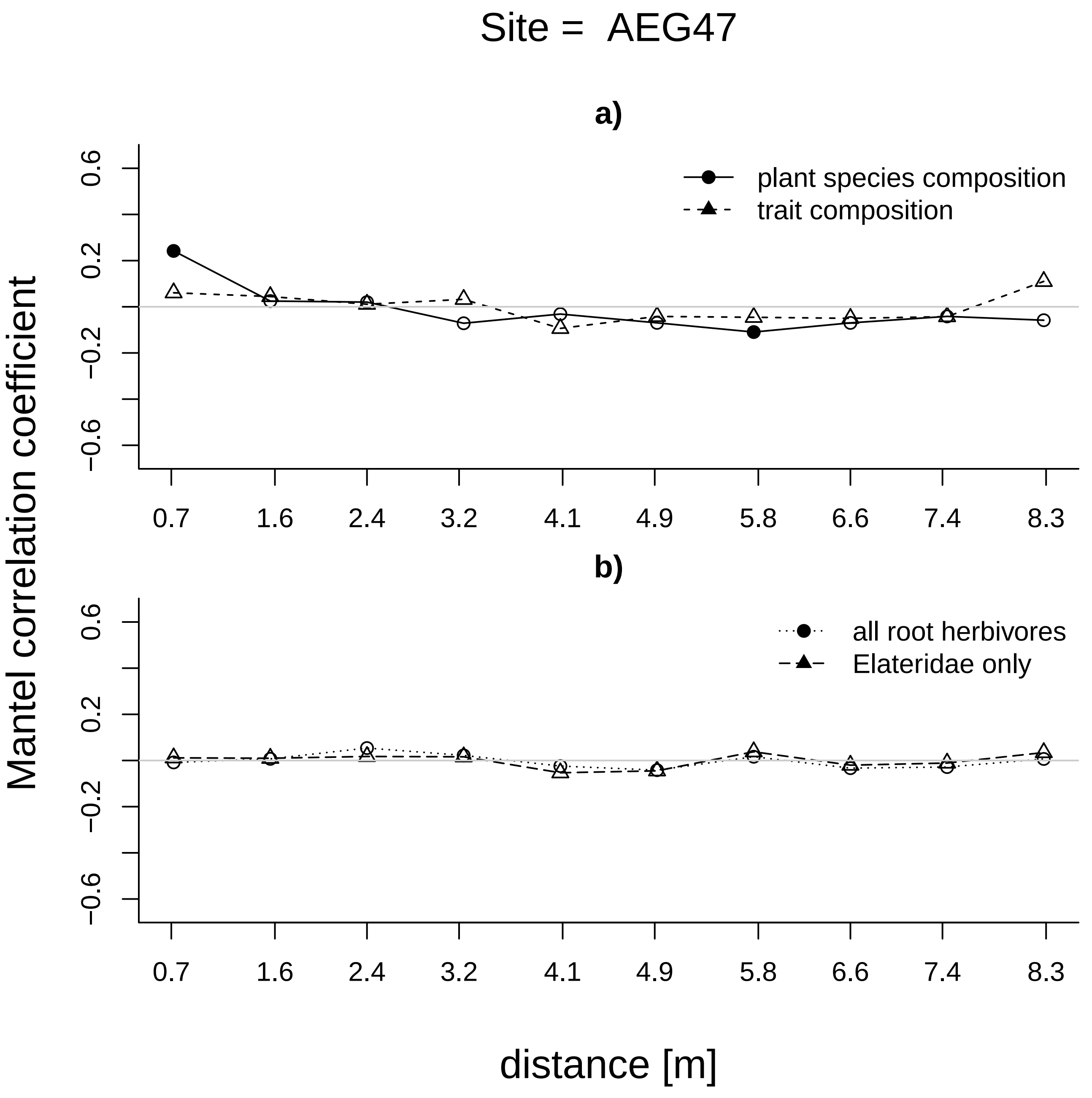
Site = AEG47
a)
plant species composition
trait composition
b)
all root herbivores
Elateridae only
Mantel correlation coefficient
distance [m]
0.6
0.2
−0.2
−0.6
0.7
1.6
2.4
3.2
4.1
4.9
5.8
6.6
7.4
8.3

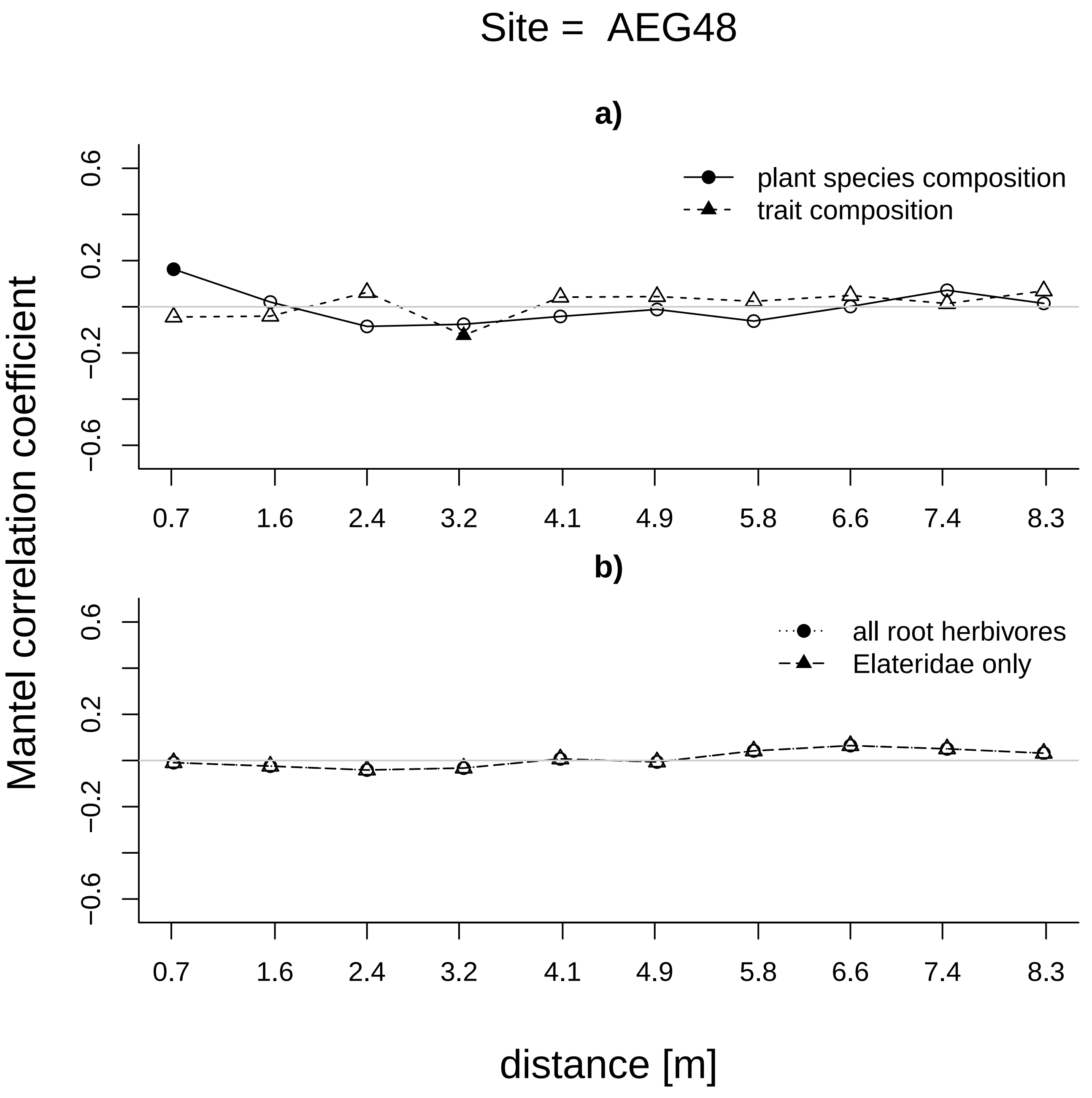
Site = AEG48
a)
plant species composition
trait composition
b)
all root herbivores
Elateridae only
Mantel correlation coefficient
0.6
0.2
−0.2
−0.6
0.7
1.6
2.4
3.2
4.1
4.9
5.8
6.6
7.4
8.3
distance [m]

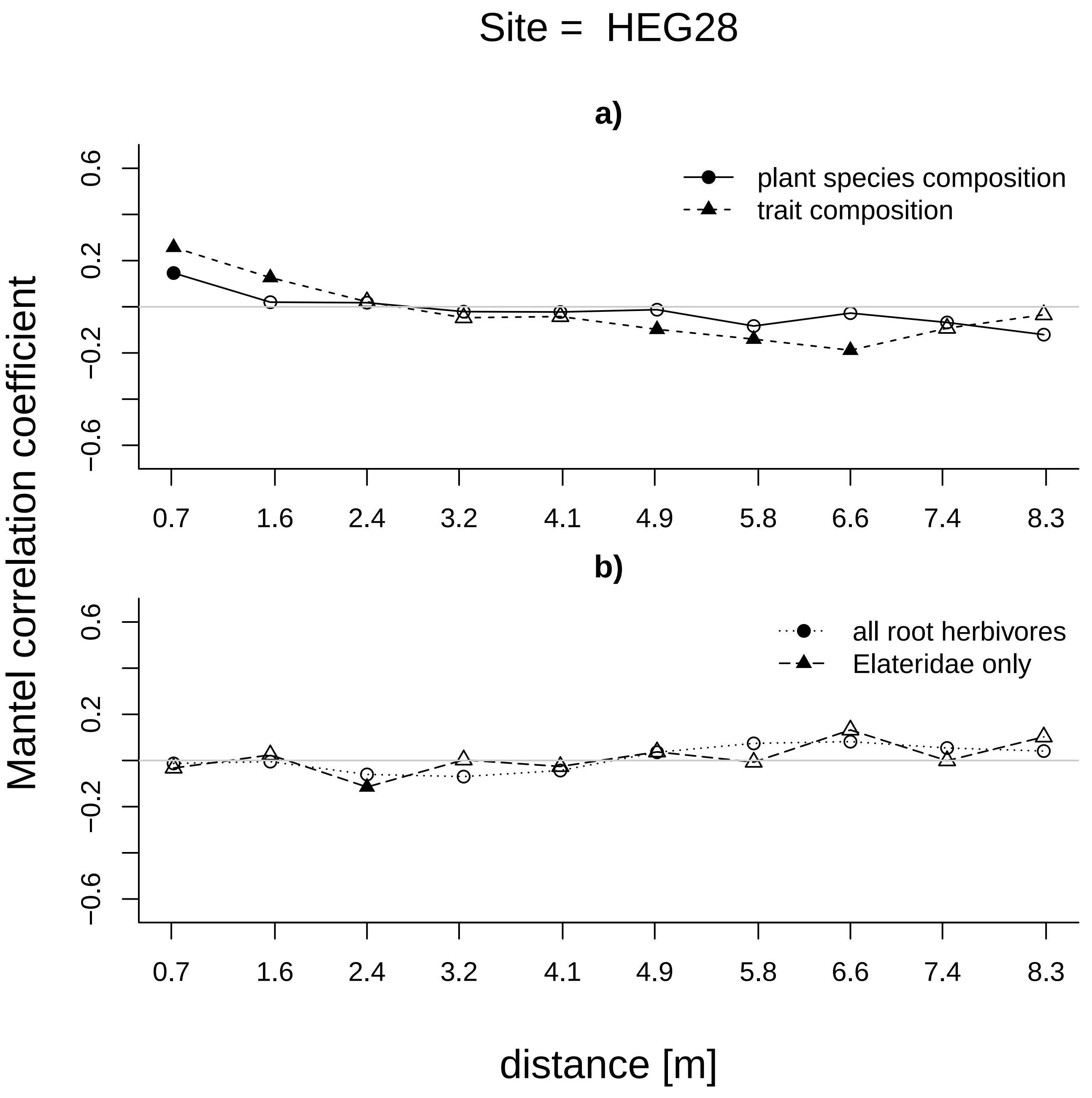

Site = HEG28
a)
plant species composition
trait composition
0.6
0.2
−0.2
−0.6
0.7
1.6
2.4
3.2
4.1
4.9
5.8
6.6
7.4
8.3
b)
all root herbivores
Elateridae only
0.6
0.2
−0.2
−0.6
0.7
1.6
2.4
3.2
4.1
4.9
5.8
6.6
7.4
8.3
Mantel correlation coefficient
distance [m]

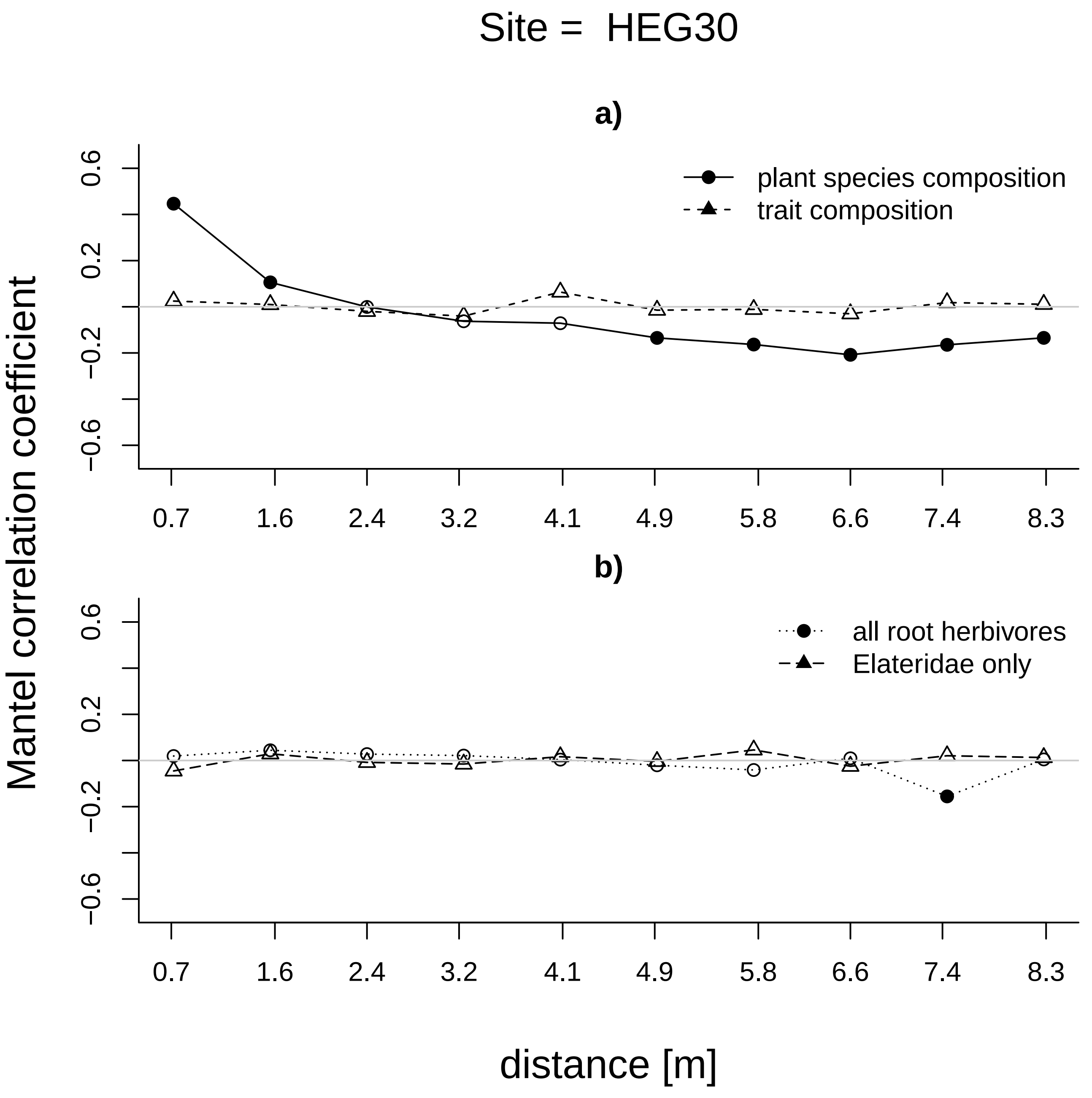

Site = HEG30
a)
plant species composition
trait composition
b)
all root herbivores
Elateridae only
Mantel correlation coefficient
0.6
0.2
−0.2
−0.6
0.7
1.6
2.4
3.2
4.1
4.9
5.8
6.6
7.4
8.3
distance [m]

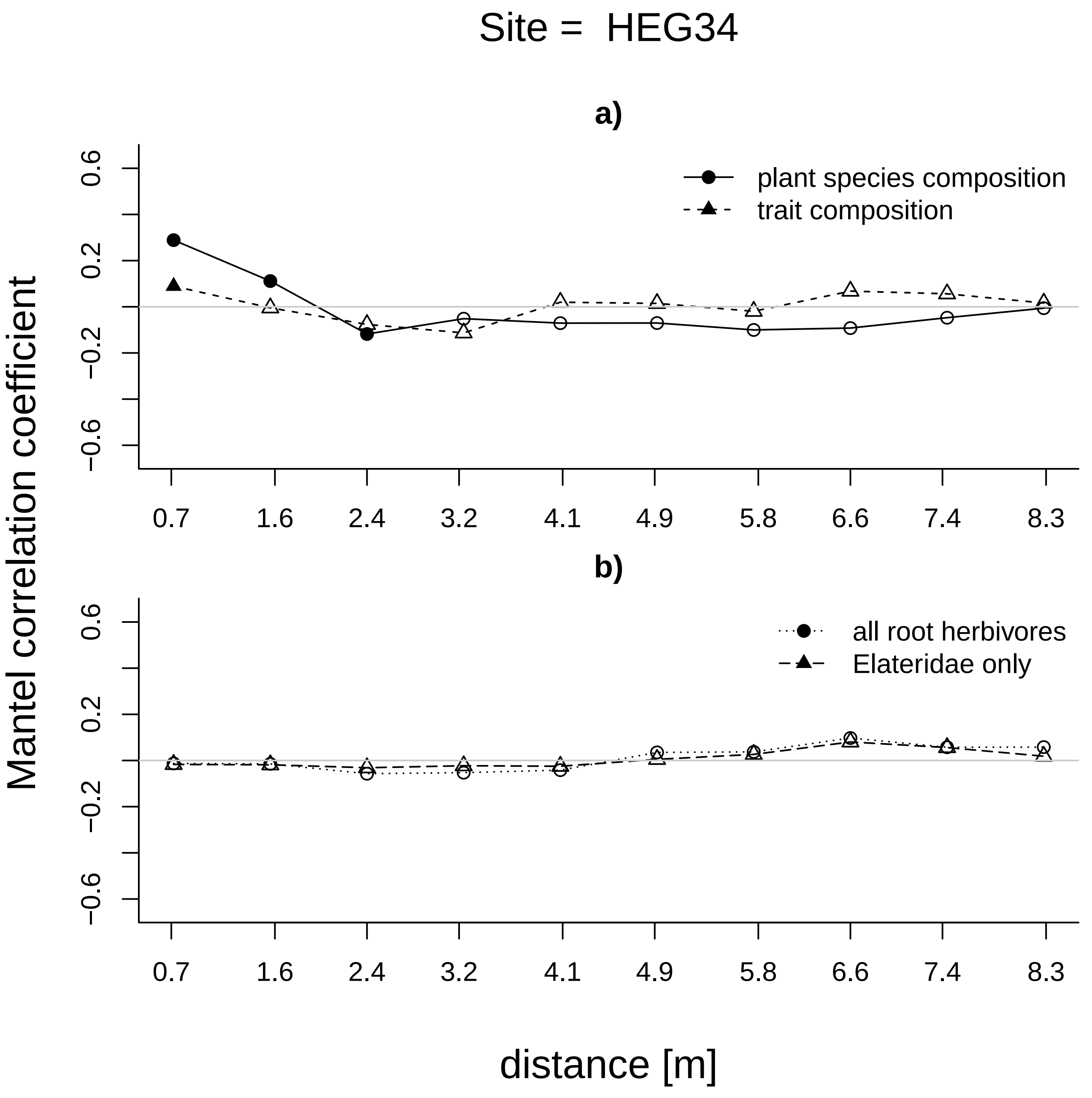
Site = HEG34
a)
plant species composition
trait composition
b)
all root herbivores
Elateridae only
Mantel correlation coefficient
0.6
0.2
−0.2
−0.6
0.7
1.6
2.4
3.2
4.1
4.9
5.8
6.6
7.4
8.3
distance [m]

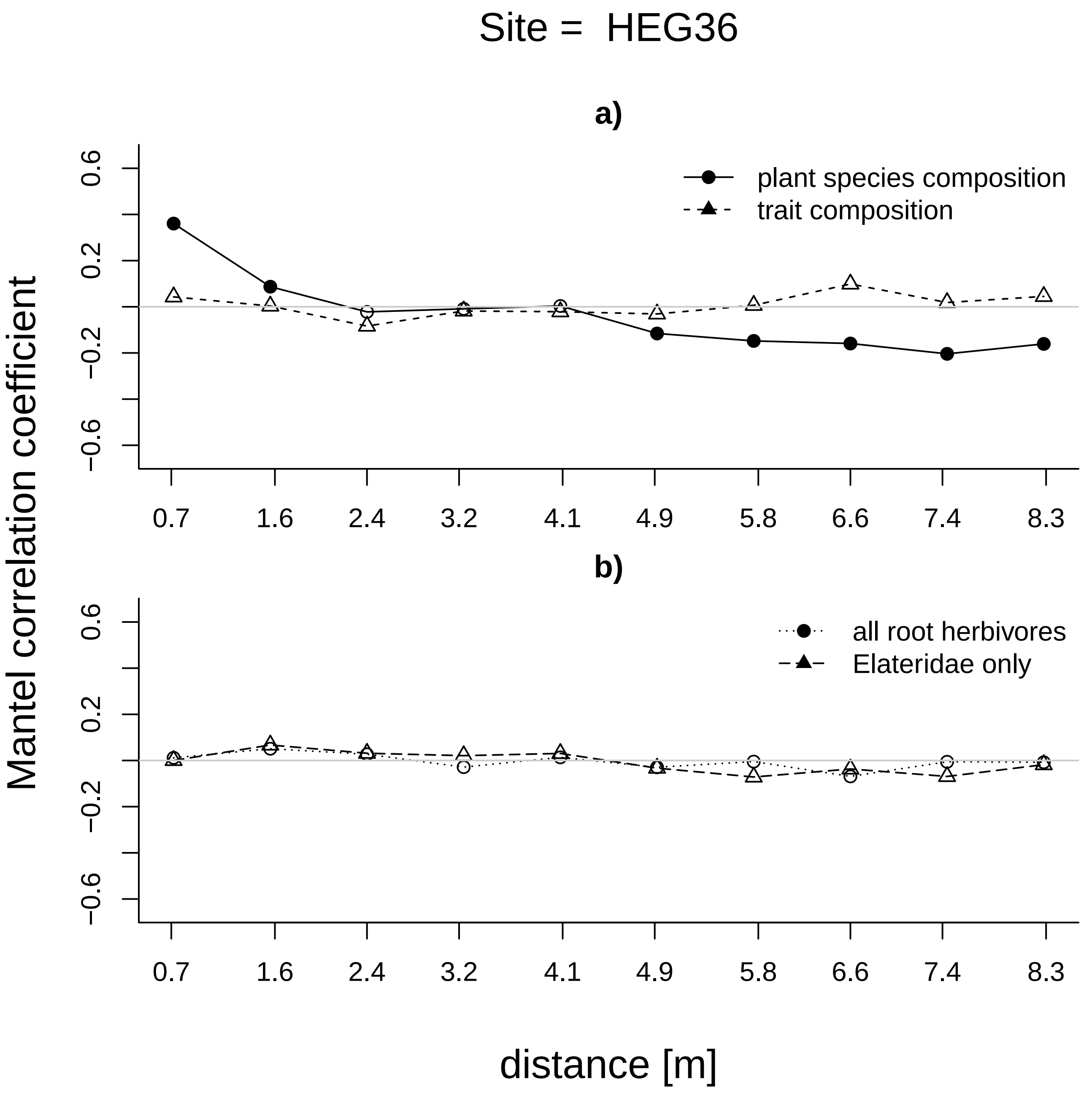
Site = HEG36
a)
plant species composition
trait composition
0.6
0.2
−0.2
−0.6
0.7
1.6
2.4
3.2
4.1
4.9
5.8
6.6
7.4
8.3
b)
all root herbivores
Elateridae only
0.6
0.2
−0.2
−0.6
0.7
1.6
2.4
3.2
4.1
4.9
5.8
6.6
7.4
8.3
Mantel correlation coefficient
distance [m]

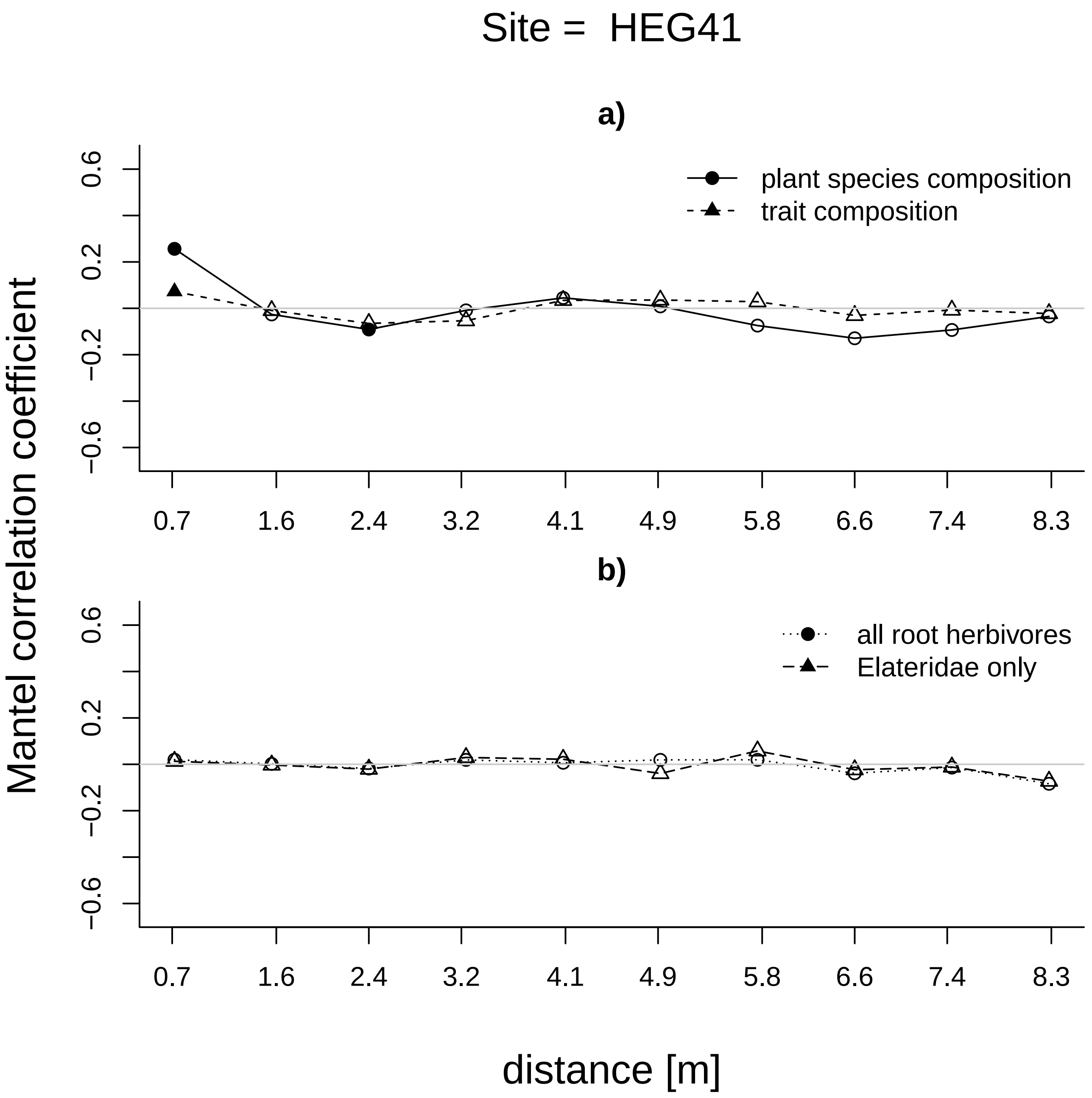

Site = HEG41
a)
plant species composition
trait composition
Mantel correlation coefficient
0.6
0.2
−0.2
−0.6
0.7
1.6
2.4
3.2
4.1
4.9
5.8
6.6
7.4
8.3
b)
all root herbivores
Elateridae only
distance [m]

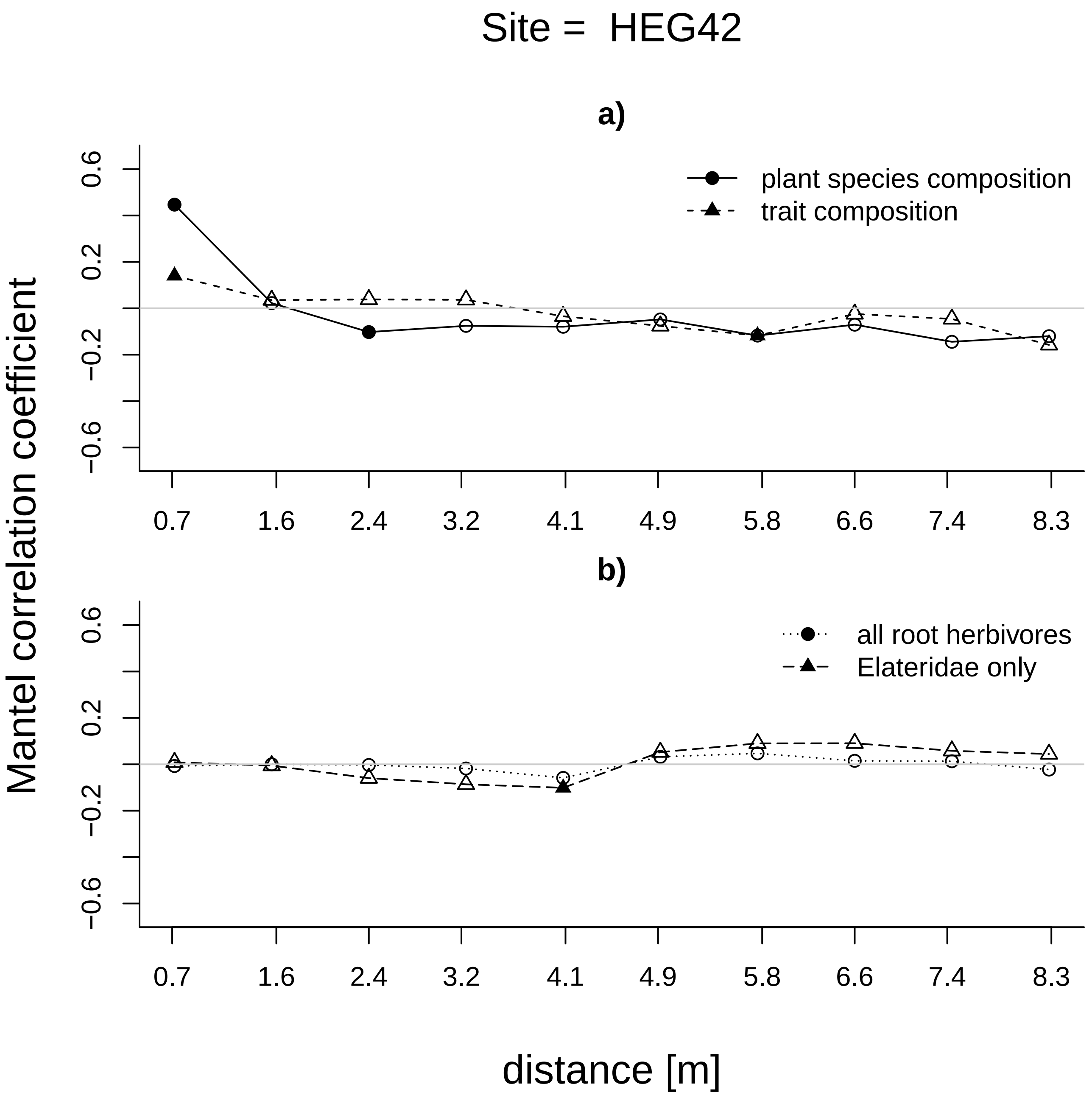

Site = HEG42
a)
plant species composition
trait composition
b)
all root herbivores
Elateridae only
Mantel correlation coefficient
0.6
0.2
−0.2
−0.6
0.7
1.6
2.4
3.2
4.1
4.9
5.8
6.6
7.4
8.3
distance [m]

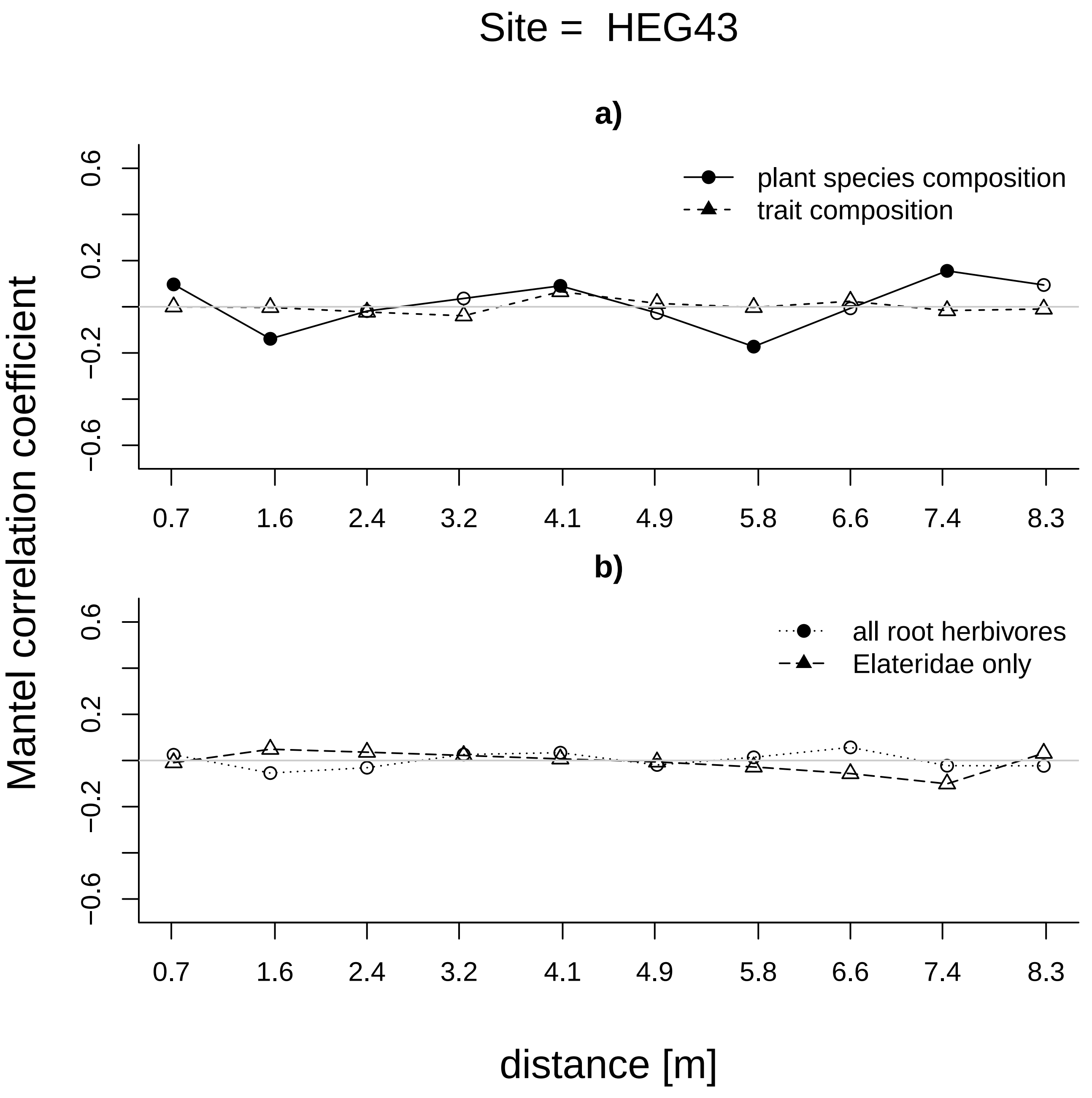

Site = HEG43
a)
plant species composition
trait composition
0.6
0.2
−0.2
−0.6
0.7
1.6
2.4
3.2
4.1
4.9
5.8
6.6
7.4
8.3
b)
all root herbivores
Elateridae only
0.6
0.2
−0.2
−0.6
0.7
1.6
2.4
3.2
4.1
4.9
5.8
6.6
7.4
8.3
Mantel correlation coefficient
distance [m]

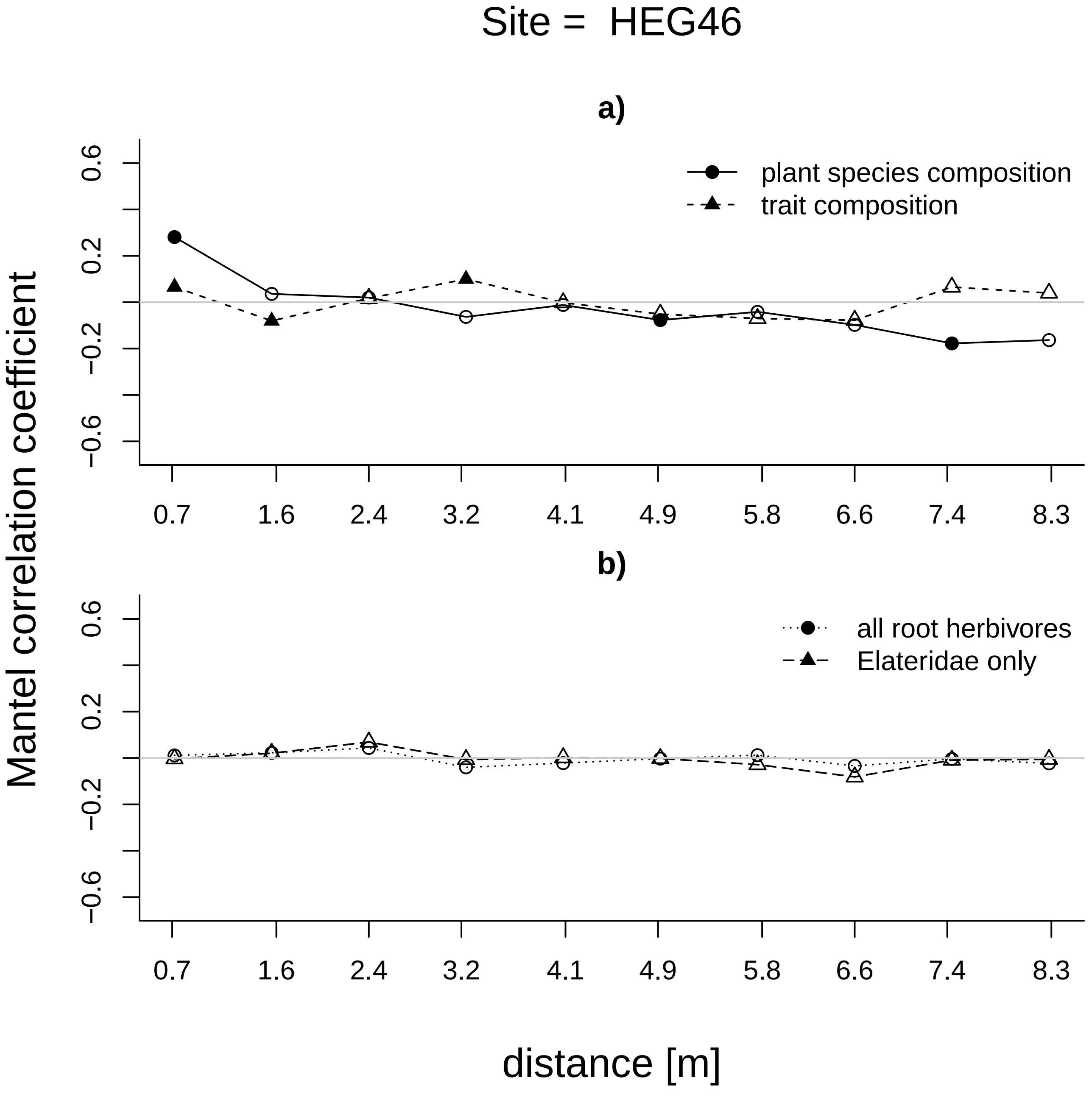

Site = HEG46
a)
plant species composition
trait composition
b)
all root herbivores
Elateridae only
Mantel correlation coefficient
0.6
0.2
−0.2
−0.6
0.7
1.6
2.4
3.2
4.1
4.9
5.8
6.6
7.4
8.3
distance [m]

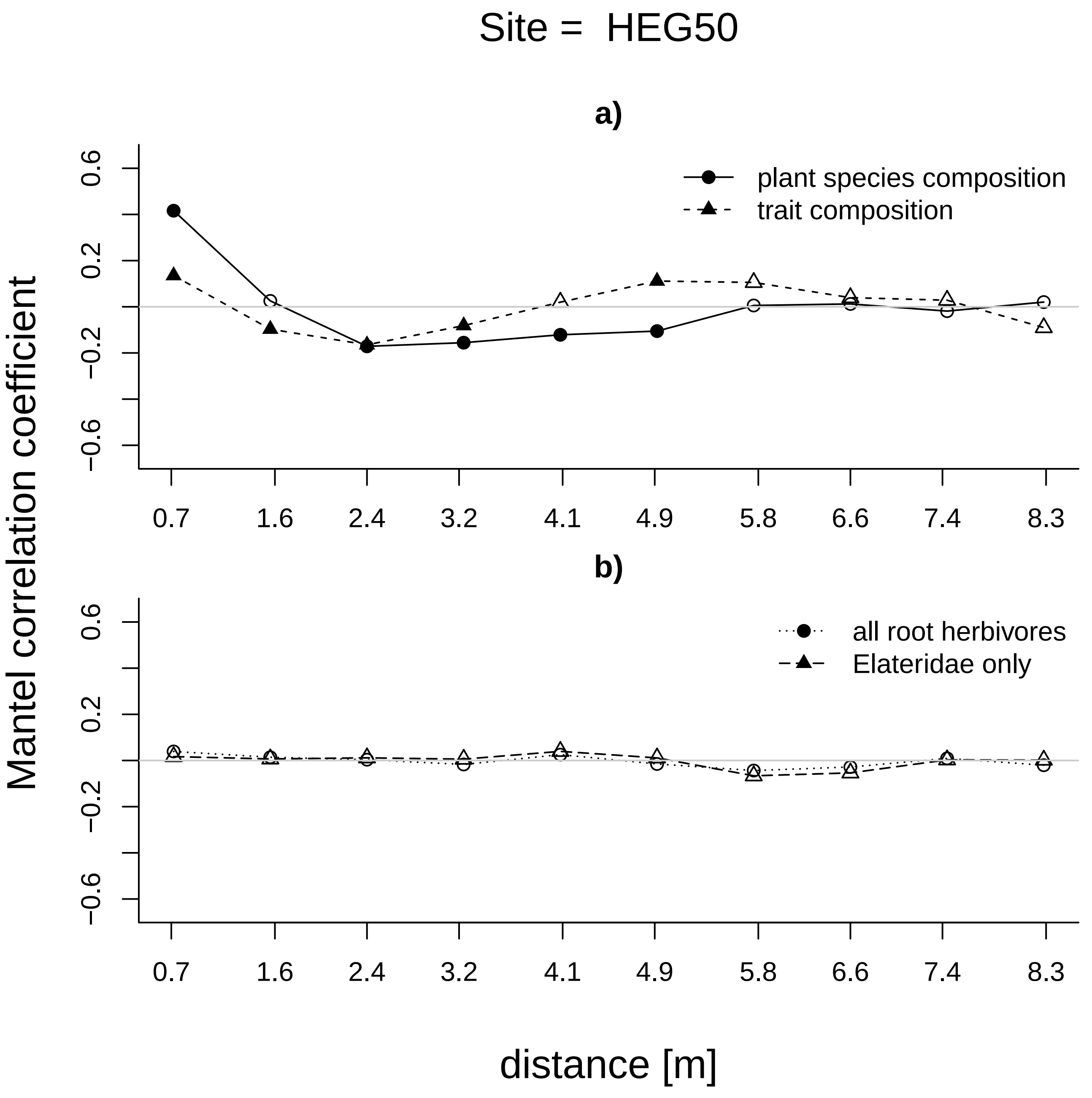

Site = HEG50
a)
plant species composition
trait composition
0.6
0.2
−0.2
−0.6
0.7
1.6
2.4
3.2
4.1
4.9
5.8
6.6
7.4
8.3
b)
all root herbivores
Elateridae only
0.6
0.2
−0.2
−0.6
0.7
1.6
2.4
3.2
4.1
4.9
5.8
6.6
7.4
8.3
Mantel correlation coefficient
distance [m]

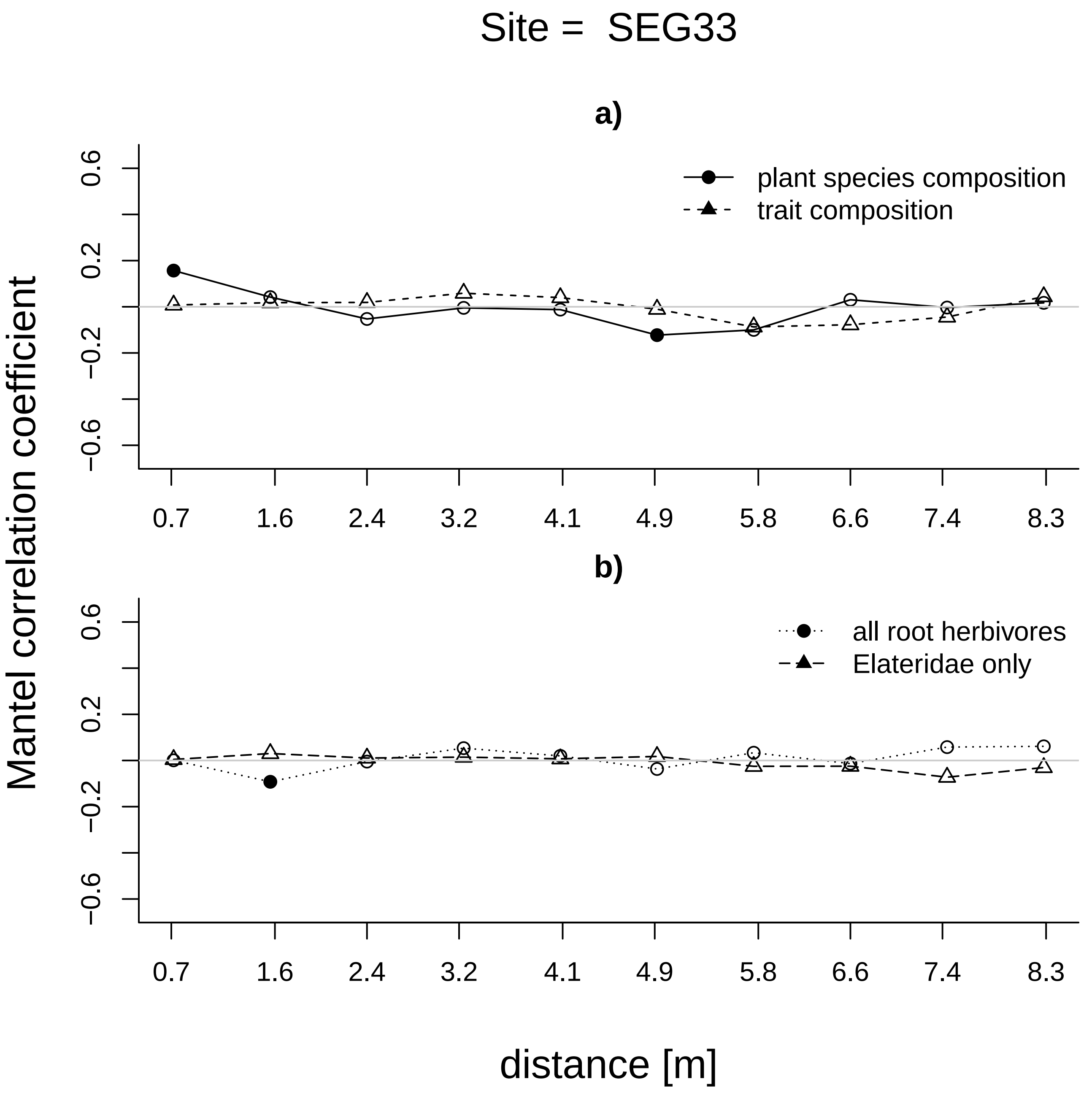

Site = SEG33
a)
plant species composition
trait composition
0.6
0.2
−0.2
−0.6
0.7
1.6
2.4
3.2
4.1
4.9
5.8
6.6
7.4
8.3
b)
all root herbivores
Elateridae only
Mantel correlation coefficient
distance [m]

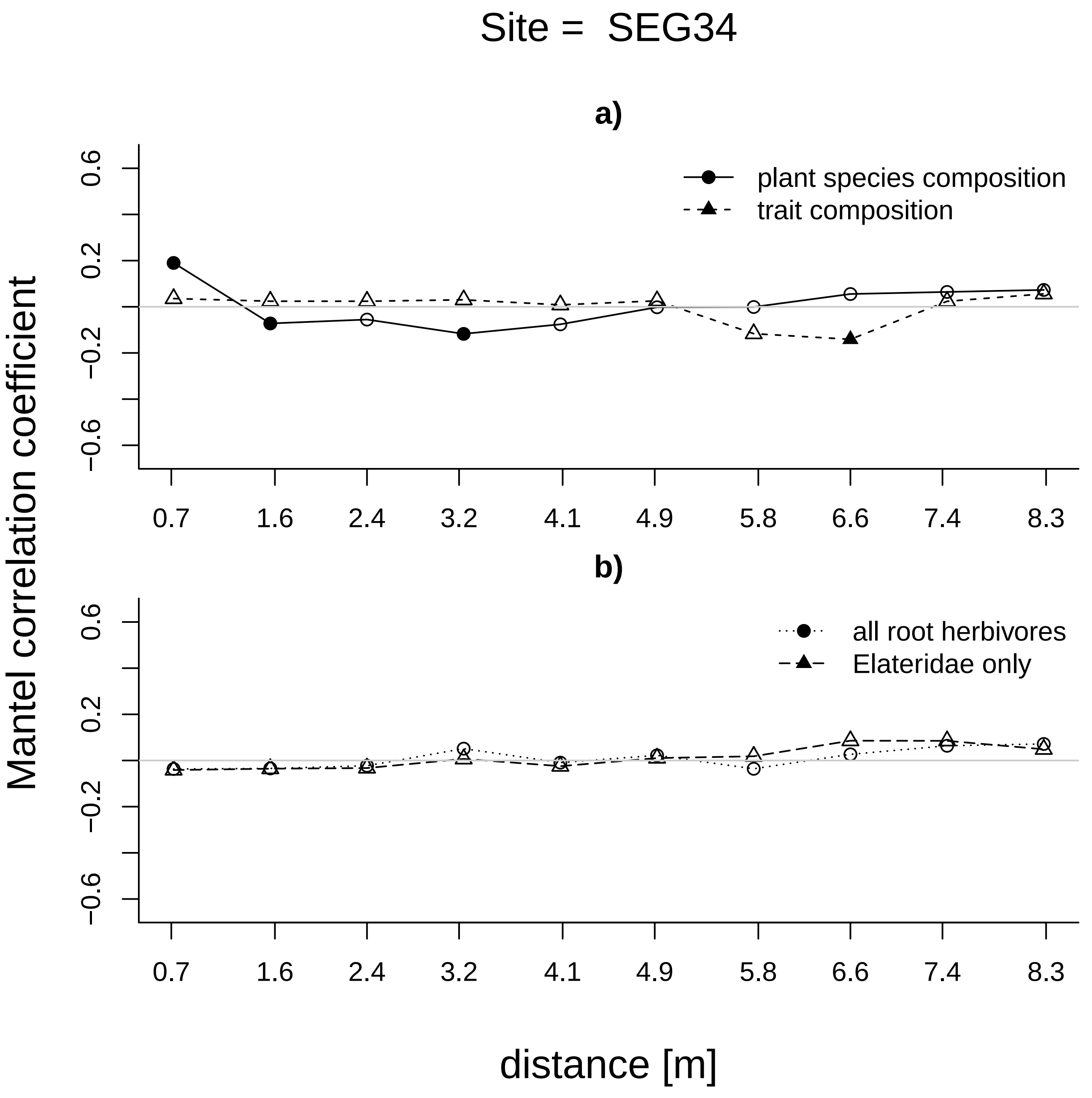

Site = SEG34
a)
plant species composition
trait composition
0.6
0.2
−0.2
−0.6
0.7
1.6
2.4
3.2
4.1
4.9
5.8
6.6
7.4
8.3
b)
all root herbivores
Elateridae only
0.6
0.2
−0.2
−0.6
0.7
1.6
2.4
3.2
4.1
4.9
5.8
6.6
7.4
8.3
Mantel correlation coefficient
distance [m]

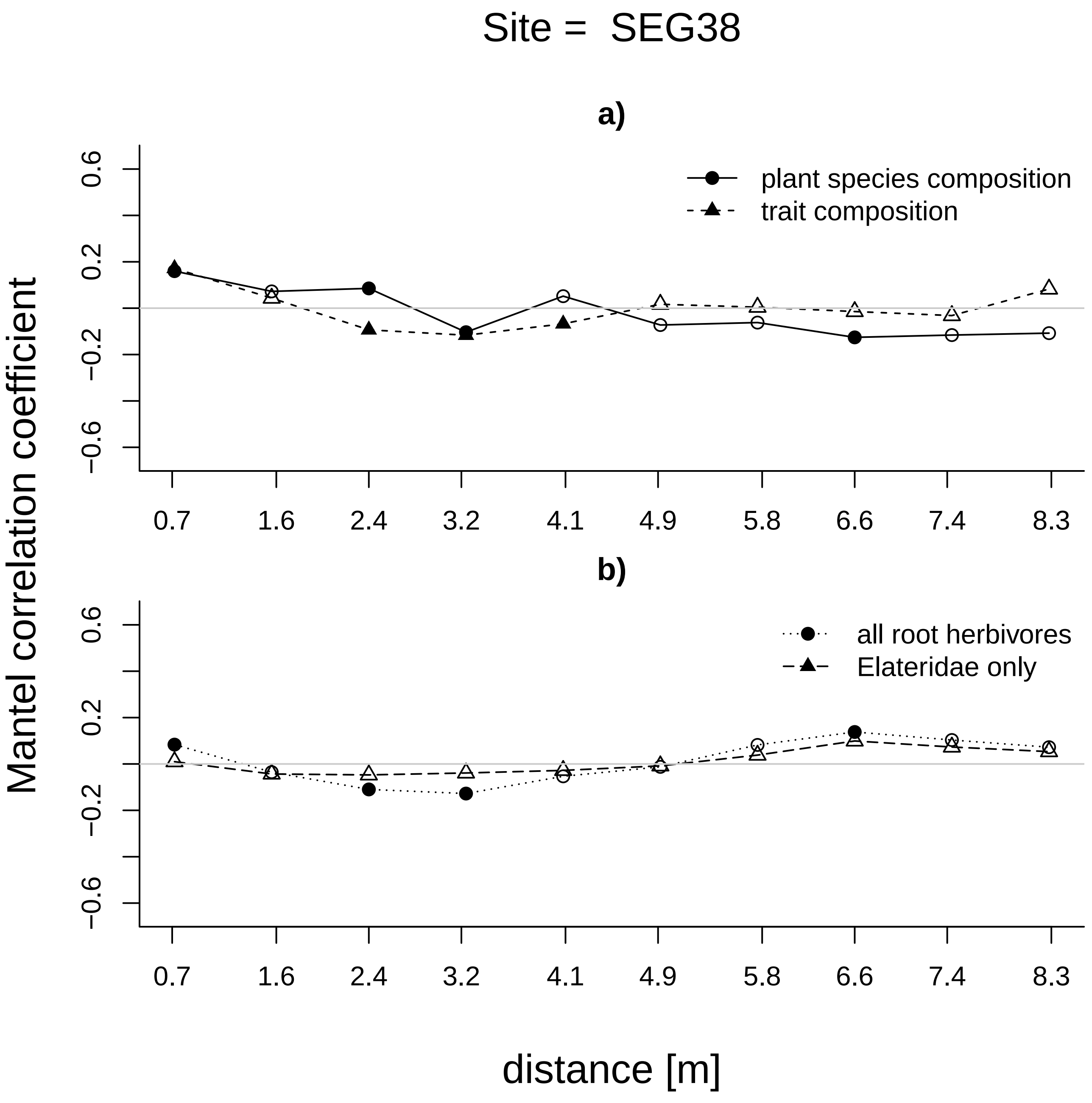

Site = SEG38
a)
plant species composition
trait composition
0.6
0.2
−0.2
−0.6
0.7
1.6
2.4
3.2
4.1
4.9
5.8
6.6
7.4
8.3
b)
all root herbivores
Elateridae only
Mantel correlation coefficient
distance [m]

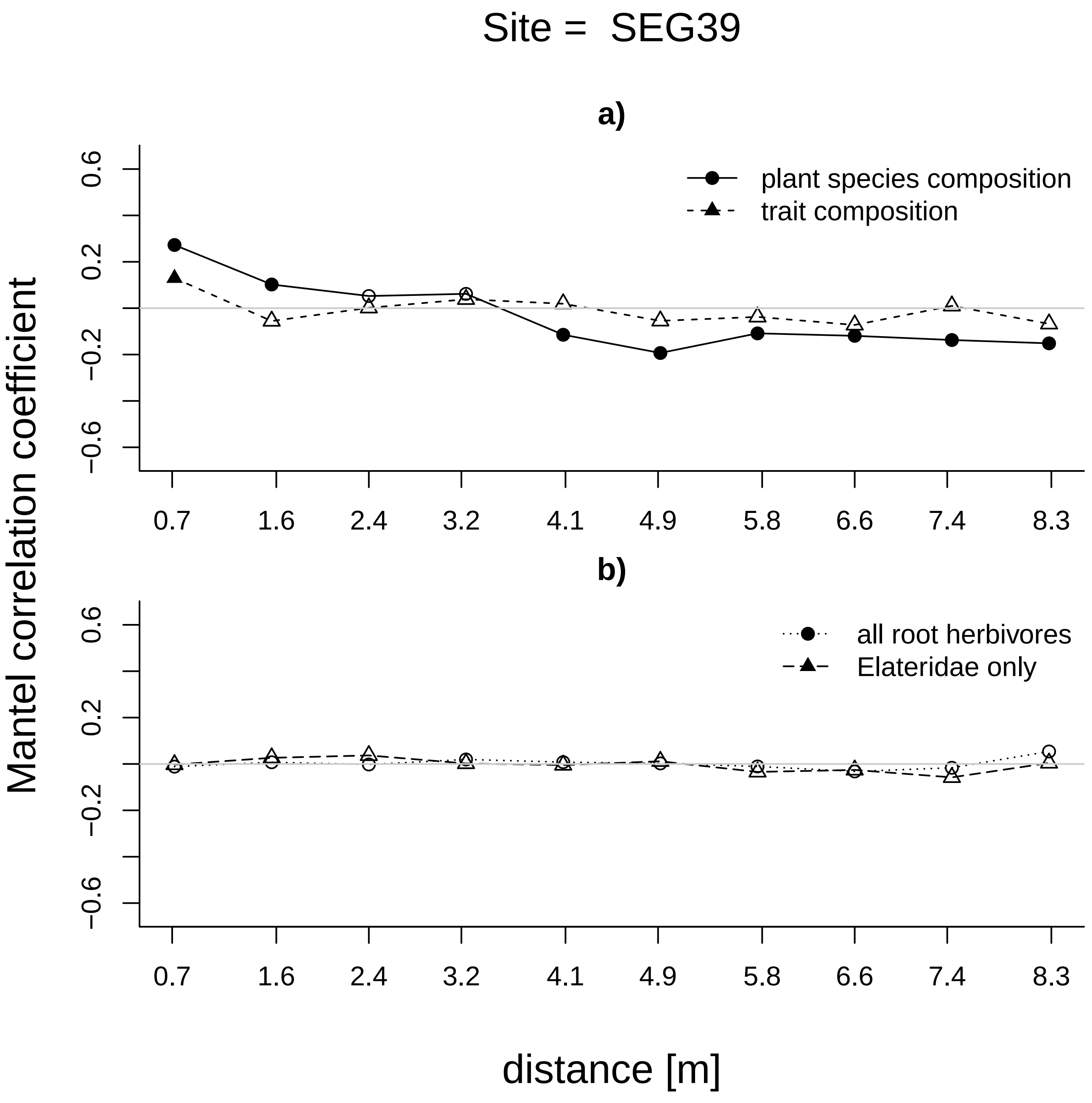

Site = SEG39
a)
plant species composition
trait composition
b)
all root herbivores
Elateridae only
Mantel correlation coefficient
0.6
0.2
−0.2
−0.6
0.7
1.6
2.4
3.2
4.1
4.9
5.8
6.6
7.4
8.3
distance [m]

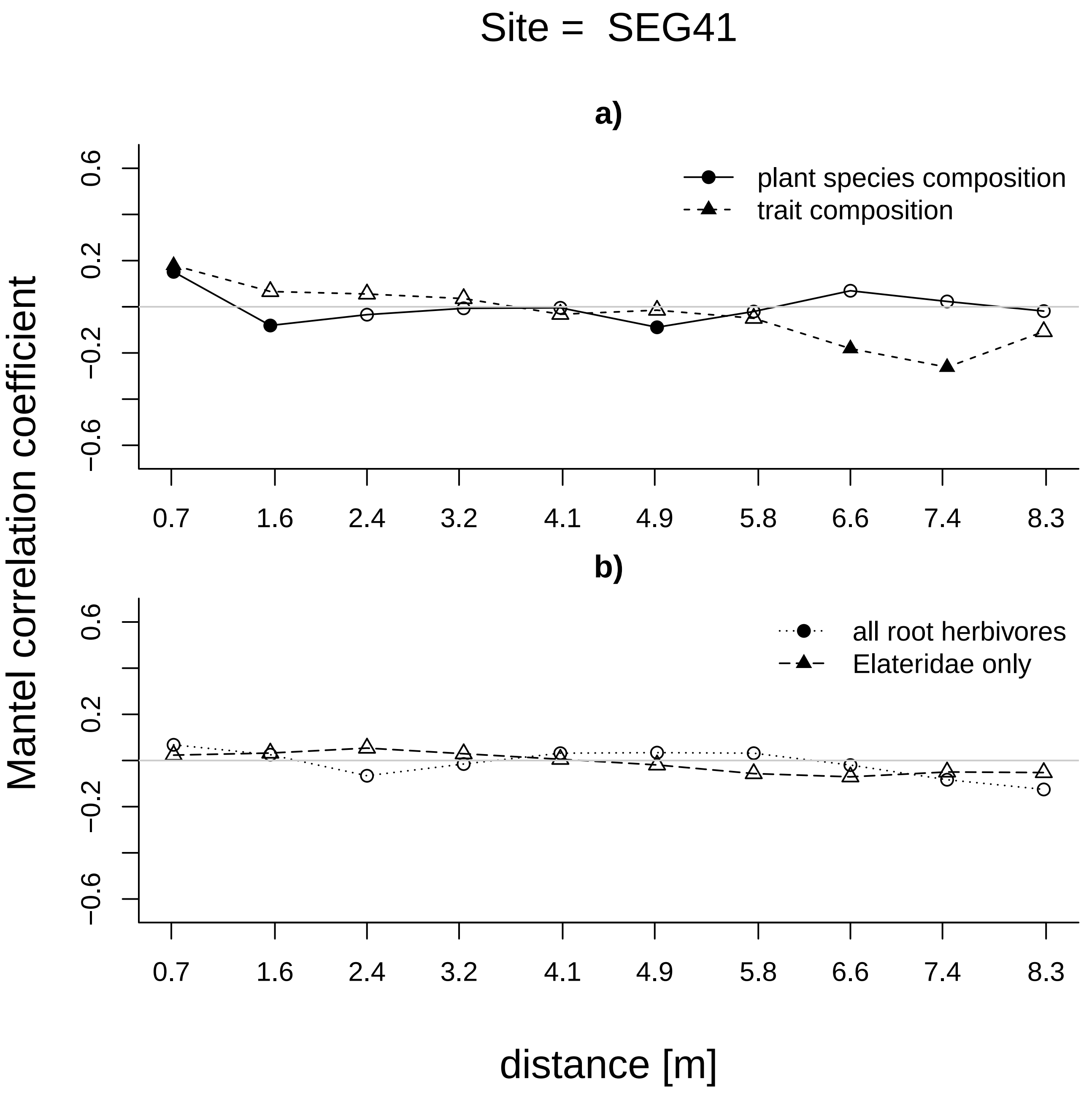

Site = SEG41
a)
plant species composition
trait composition
0.6
0.2
−0.2
−0.6
0.7
1.6
2.4
3.2
4.1
4.9
5.8
6.6
7.4
8.3
b)
all root herbivores
Elateridae only
Mantel correlation coefficient
distance [m]

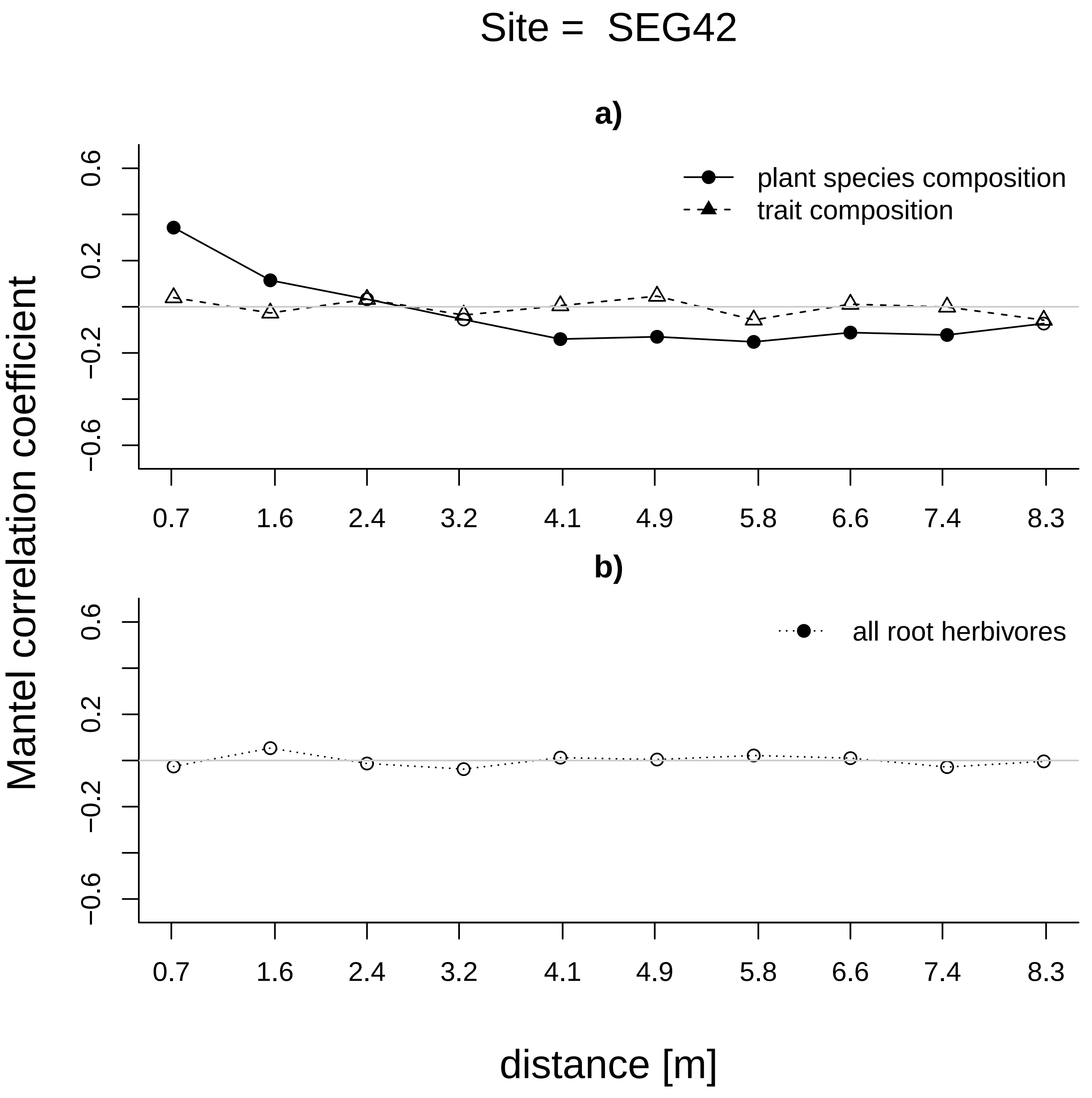
Site = SEG42
a)
plant species composition
trait composition
0.6
0.2
−0.2
−0.6
0.7
1.6
2.4
3.2
4.1
4.9
5.8
6.6
7.4
8.3
b)
all root herbivores
Mantel correlation coefficient
distance [m]

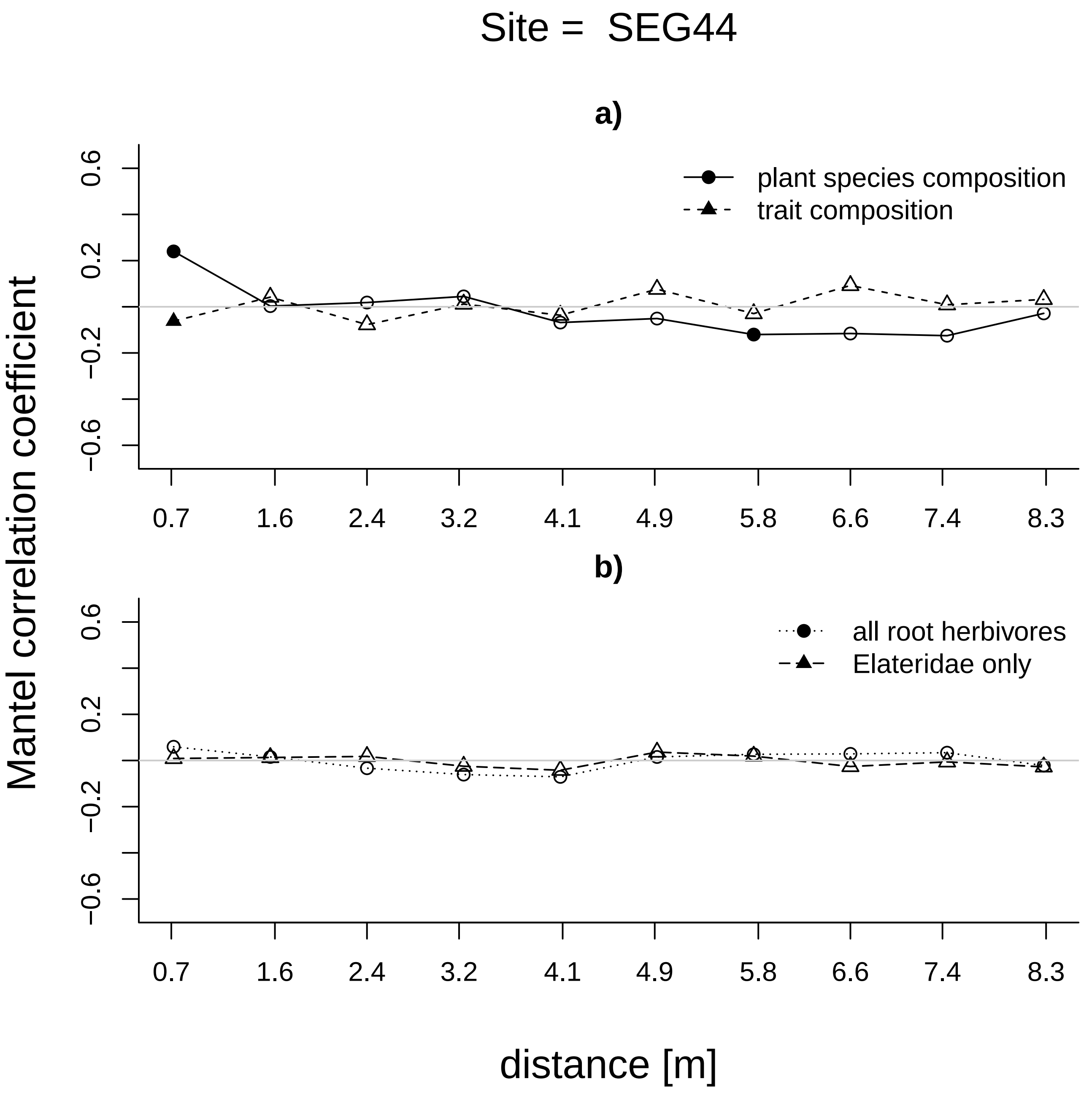

Site = SEG44
a)
plant species composition
trait composition
0.6
0.2
−0.2
−0.6
0.7
1.6
2.4
3.2
4.1
4.9
5.8
6.6
7.4
8.3
b)
all root herbivores
Elateridae only
Mantel correlation coefficient
distance [m]

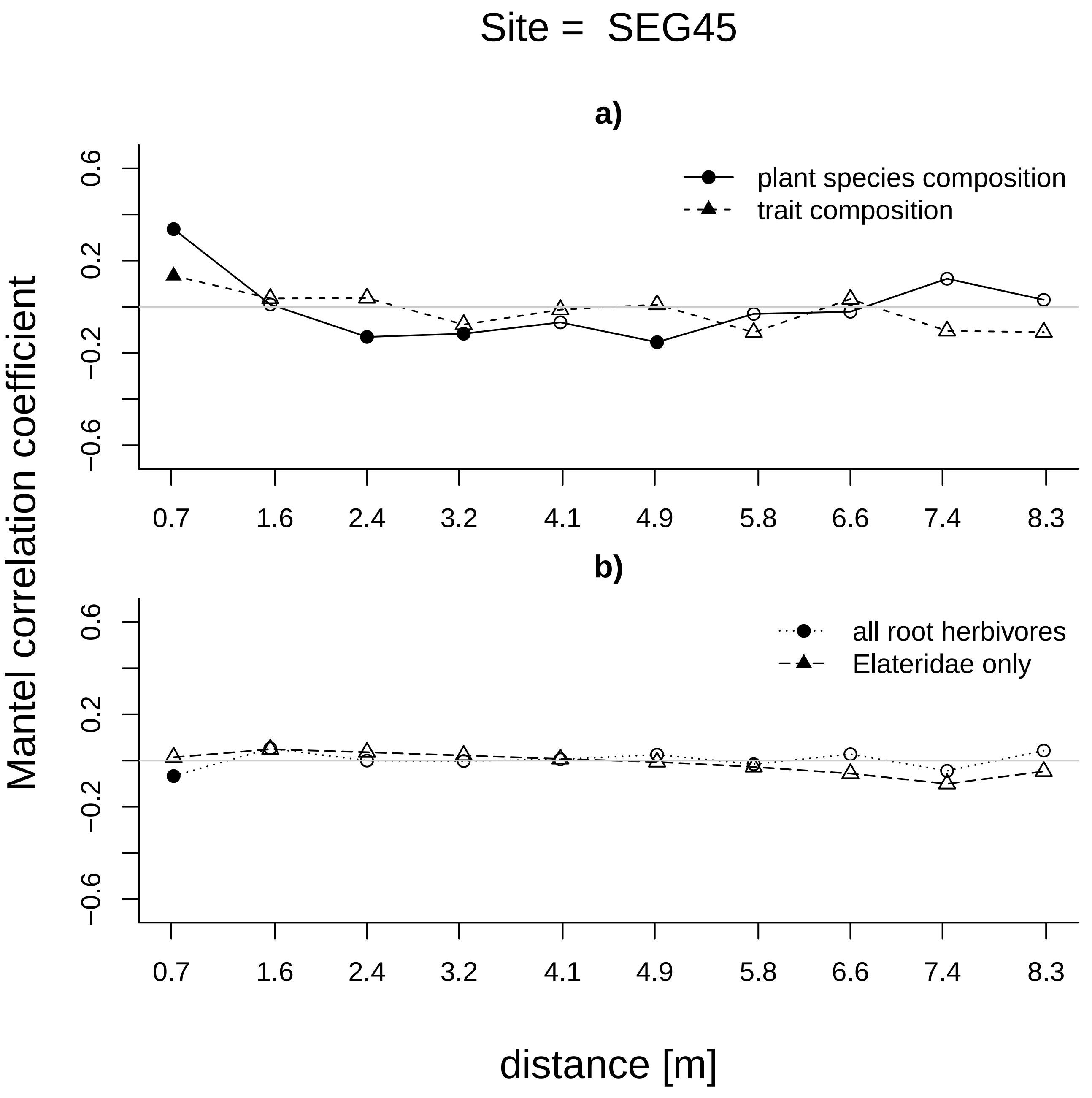
Site = SEG45
a)
plant species composition
trait composition
b)
all root herbivores
Elateridae only
Mantel correlation coefficient
0.6
0.2
−0.2
−0.6
0.7
1.6
2.4
3.2
4.1
4.9
5.8
6.6
7.4
8.3
distance [m]

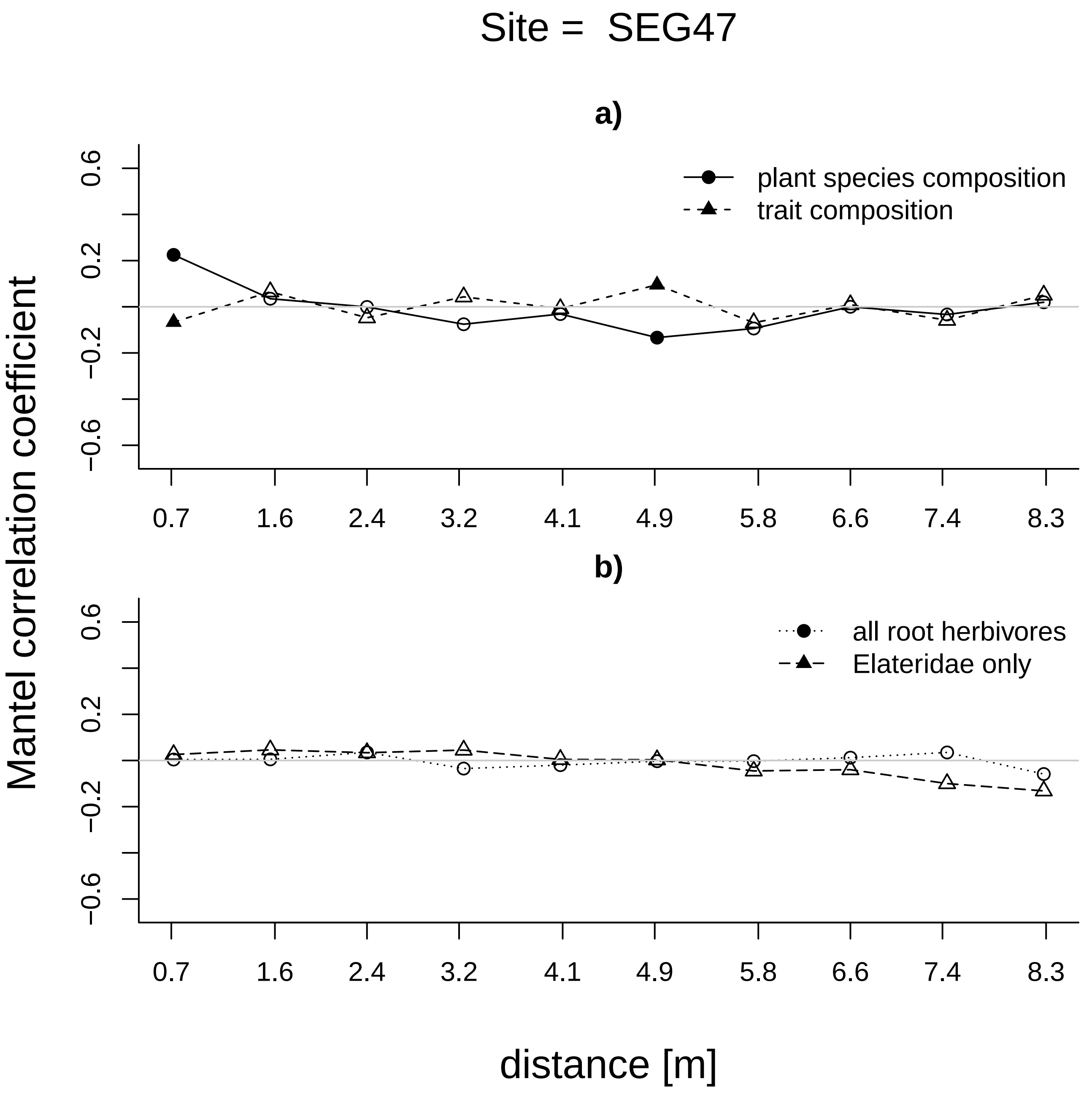
Site = SEG47
a)
plant species composition
trait composition
Mantel correlation coefficient
0.6
0.2
−0.2
−0.6
0.7
1.6
2.4
3.2
4.1
4.9
5.8
6.6
7.4
8.3
b)
all root herbivores
Elateridae only
0.6
0.2
−0.2
−0.6
0.7
1.6
2.4
3.2
4.1
4.9
5.8
6.6
7.4
8.3
distance [m]

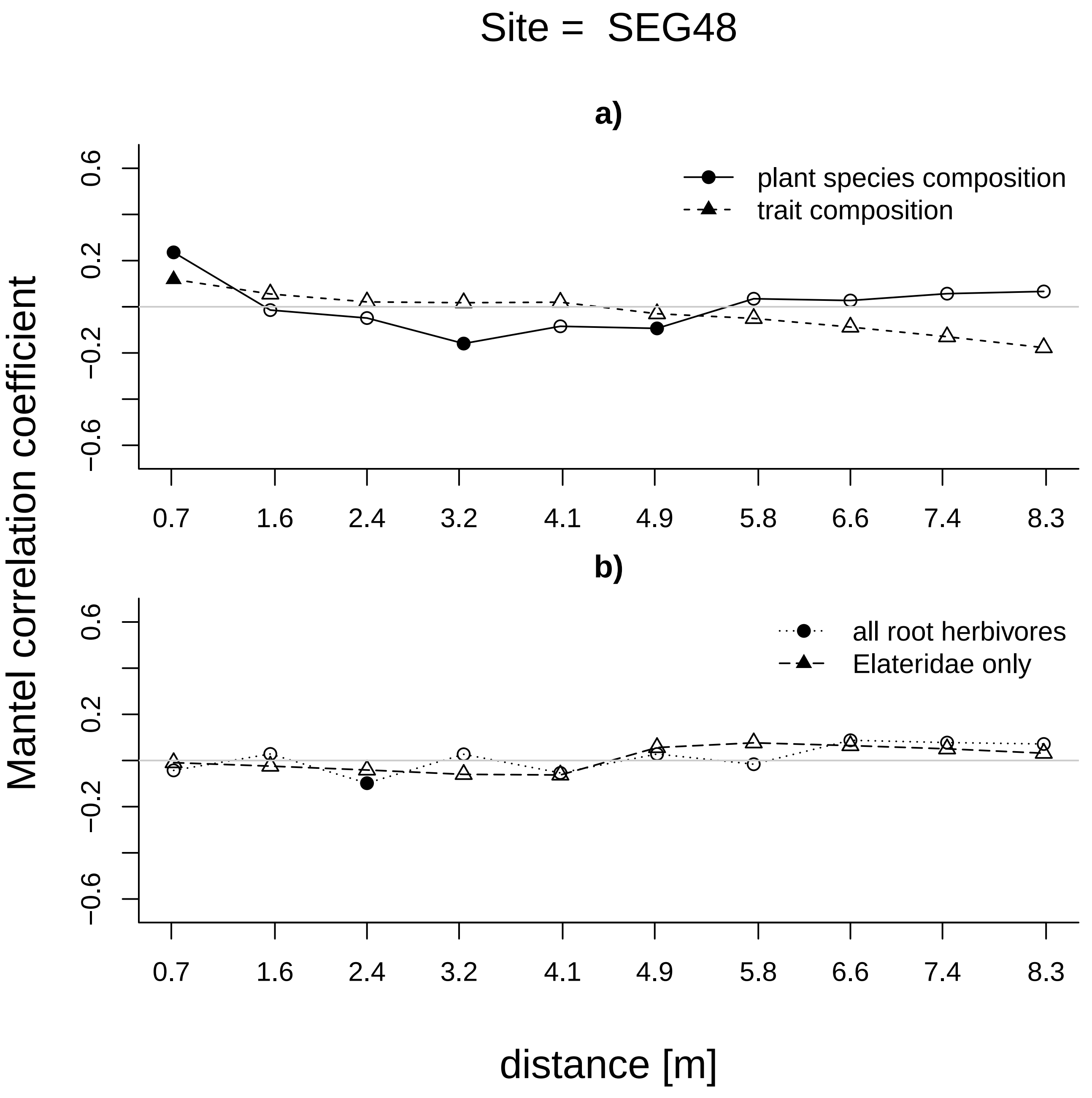
Site = SEG48
a)
plant species composition
trait composition
b)
all root herbivores
Elateridae only
Mantel correlation coefficient
0.6
0.2
−0.2
−0.6
0.7
1.6
2.4
3.2
4.1
4.9
5.8
6.6
7.4
8.3
distance [m]
