# Supplementary material for: Community- Weighted Mean Plant Traits Predict Small Scale Distribution of Insect Root Herbivore Abundance
Source: PLoS One. 2015 Oct 30;10(10):e0141148. doi: 10.1371/journal.pone.0141148 (PMC4627808; doi:10.1371/journal.pone.0141148)
Supplement: S2 Table — (DOCX) [file pone.0141148.s003.docx]

|  | MCC  plant species composition | | MCC  plant trait composition | |
| --- | --- | --- | --- | --- |
| **Predictor** | **χ²** | **p** | **χ²** | **p** |
| LUI | 0.33 | ns | 0.01 | ns |
| WHC | 0.27 | ns | 0.35 | ns |
| Distance | 34.60 | ** | 7.51 | ns |
| LUI x WHC | 0.11 | ns | 0.00 | ns |
| LUI x distance | 8.72 | ns | 1.79 | ns |
| WHC x distance | 12.08 | ns | 5.56 | ns |
| Significance levels; **: p<0.01, *: p<0.05, ns: not significant p>0.05 | | | | |
